# Drug Delivery Nanoparticles in Skin Cancers

**DOI:** 10.1155/2014/895986

**Published:** 2014-07-02

**Authors:** Chiara Dianzani, Gian Paolo Zara, Giovanni Maina, Piergiorgio Pettazzoni, Stefania Pizzimenti, Federica Rossi, Casimiro Luca Gigliotti, Eric Stefano Ciamporcero, Martina Daga, Giuseppina Barrera

**Affiliations:** ^1^Department of Drug Science and Technology, University of Turin, 10125 Turin, Italy; ^2^Department of Clinical and Biological Sciences, University of Turin, 10125 Turin, Italy; ^3^Department of Genomic Medicine, University of Texas MD Anderson Cancer Center, Houston, TX 77030, USA; ^4^Department of Public and Pediatric Health Sciences, University of Turin, 10126 Turin, Italy; ^5^Department of Health Sciences, University of Eastern Piedmont “Amedeo Avogadro,” 28100 Novara, Italy

## Abstract

Nanotechnology involves the engineering of functional systems at nanoscale, thus being attractive for disciplines ranging from materials science to biomedicine. One of the most active research areas of the nanotechnology is nanomedicine, which applies nanotechnology to highly specific medical interventions for prevention, diagnosis, and treatment of diseases, including cancer disease. Over the past two decades, the rapid developments in nanotechnology have allowed the incorporation of multiple therapeutic, sensing, and targeting agents into nanoparticles, for detection, prevention, and treatment of cancer diseases. Nanoparticles offer many advantages as drug carrier systems since they can improve the solubility of poorly water-soluble drugs, modify pharmacokinetics, increase drug half-life by reducing immunogenicity, improve bioavailability, and diminish drug metabolism. They can also enable a tunable release of therapeutic compounds and the simultaneous delivery of two or more drugs for combination therapy. In this review, we discuss the recent advances in the use of different types of nanoparticles for systemic and topical drug delivery in the treatment of skin cancer. In particular, the progress in the treatment with nanocarriers of basal cell carcinoma, squamous cell carcinoma, and melanoma has been reported.

## 1. Introduction 

Nanotechnology is a generalization for techniques, materials, and equipment that operate at the nanoscale. It is a revolutionary approach that consists of the design, characterization, preparation, and application of structures, devices, and systems by controlling shape and size at the nanoscale [1]. According to the federal US research and development program agency, the National Nanotechnology Initiative (NNI), nanotechnology involves the development of carriers devices or systems sized in 1 to 100 nm range although this limit can be extended up to 1000 nm [2]. These biomimetic features, together with their high surface-to-volume ratio and the possibility of modulating their properties, raised the interest of the use in biomedical application with potential applications in imaging, diagnosis, and therapy [3].

Over the past two decades, the rapid developments in nanotechnology have allowed the incorporation of multiple therapeutic, sensing, and targeting agents into nanoparticles, for detection, prevention, and treatment of oncologic diseases.

Nanomedicine has an enormous potential to improve the selectivity in targeting neoplastic cells by allowing the preferential delivery of drugs to tumours owing to the enhanced permeability and retention effect (EPR). Furthermore, specific binding of drugs to targets in cancer cells or the tumour microenvironment increases the effectiveness of the specific treatment of cancer cells, while leaving healthy cells intact. Nanoparticles (NP) can also improve the solubility of poorly water-soluble drugs, modify pharmacokinetics, increase drug half-life by reducing immunogenicity, improve bioavailability, and diminish drug metabolism. They can also enable a tunable release of therapeutic compounds and the simultaneous delivery of two or more drugs for combination therapy [4, 5]. In addition, by reducing the drug doses, it is also possible to reduce side effects and ameliorate the patients' compliance [6]. These engineered nanocarriers offer also the opportunity to use the combination of imaging and drug therapy to monitor effects in real time, as well as the possibility to join the delivery of drug with energy (heat, light, and sound) for synergistic anticancer therapeutic effects [7].

Although skin cancer is not the most mortal form of cancers, it is the most common form of malignancy in the United States and many other countries [8]. Melanoma represents only a very small proportion of skin cancer incidence, but it accounts for the vast majority of skin cancer deaths. Indeed, at the early stage, melanoma can be surgically removed, with a survival rate of 99%, while metastasized melanoma causes the death of 80% of patients within 5 years from the diagnosis [9]. Other types of skin cancers, basal cell carcinoma and squamous cell carcinoma, are the most common diseases. Excision is the gold standard treatment for these localized diseases. However, in very rare cases, they can diffuse to regional lymph nodes and distant sites. For metastasized skin cancers, nanoparticles provide an effective drug delivery system, allowing anticancer drugs to reach the cancer site specifically and, thus, greatly improve treatment efficacy. In the following sections, we illustrated the major forms of nanoparticles which have been used for systemic and transdermal drug delivery in skin cancers and the specific drug-nanoparticles formulations which have been reported for the treatment of basal cell carcinoma, squamous cell carcinoma, and melanoma.

## 2. Chemicophysical Characteristics of Nanoparticles Employed for Drug Delivery in Skin Cancers 

Many nanoparticles have been studied for the treatment of skin cancers, especially in melanoma treatment, including liposomes, dendrimers, polymersomes, carbon-based nanoparticles, inorganic nanoparticles, and protein-based nanoparticles. In the following paragraphs, the characteristics of the common nanoparticles used in skin cancer treatment are described.

### 2.1. Liposomes

Liposomes are phospholipid vesicles (dimension of 50–100 nm and even larger) that have a bilayered membrane structure, similar to that of biological membranes, together with an internal aqueous phase. Liposomes are classified according to size and number of layers into multi-, oligo-, or unilamellar. The aqueous core can be used for encapsulation of water-soluble drugs, whereas the lipid bilayers may retain hydrophobic or amphiphilic compounds. To escape from reticuloendothelial system (RES) uptake after i.v. injection, PEGylated liposomes, “stealth liposomes,” were developed for reducing clearance and prolonging circulation half-life [10]. Liposomes show excellent circulation, penetration, and diffusion properties. The possibility to link the liposomes surface with ligands and/or polymers increases significantly the drug delivery specificity [11]. Early research demonstrated that liposomes remain in the tumour interstitial fluid just near the tumour vessels [12]. Currently, several liposomal formulations in the clinical practice contain several drugs for the treating of different types of cancer, including melanoma [13]. Several other liposomal chemotherapeutic drugs are at the various stages of clinical trials. Moreover, advances with cationic liposomes led to the successful delivery of small interfering RNA (siRNA) [14]. New opportunities were proposed by Muthu and Feng [15] that developed theranostic liposomes, with the possibility of loading a wide variety of diagnostic NP along with anticancer drug in combination with vitamin E TPGS coating. Liposomes can also be modified to incorporate a magnetic element for use in monitoring their movement within the body using MRI [16] or to entrap gases and drugs for ultrasound-controlled drug delivery [17].

### 2.2. Solid Lipid Nanoparticles (SLNs)

SLNs were introduced at the beginning of the 1990s as an alternative delivery system to liposomes, emulsion, and polymeric NP. SLNs present a high physical stability; that is, they can protect the drugs against degradation, and they allow an easy control the drug release. The preparation of SLNs does not require the use of organic solvents. They are biodegradable and biocompatible and have low toxicity. In addition, the production and sterilization on a large scale are rather easy [18]. Solid lipid nanoparticles (SLNs) containing docetaxel improve the efficacy of this chemotherapeutic agent in colorectal (C-26) and malignant melanoma (A-375) cell lines in “in vitro” and “in vivo” experiments [19]. Cholesteryl butyrate solid lipid nanoparticles have been shown to inhibit human umbilical vein endothelial cells' adhesiveness to cancer cell lines derived from human colon-rectum, breast, prostate cancers, and melanoma [20].

### 2.3. Polymeric Micelles and Nanospheres

Polymeric micelles are formed by two or more polymer chains with different hydrophobicity. These copolymers spontaneously assemble into a core-shell micellar structure. Specifically, the hydrophobic blocks form the core in order to minimize their exposure to aqueous surroundings, whereas the hydrophilic blocks form the corona-like shell to stabilize the core through direct contact with water [21]. The typical size of micelles for pharmaceutical applications ranges from 10 to 80 nm. Micelles, being smaller than liposomes, have a short circulation time, but they show a superior uptake by tumors, because of the EPR effect. Poorly soluble drugs, with high loading capacity (5–25 wt %) can be carried in the hydrophobic core, while the hydrophilic shell allows a steric protection for the micelle and thereby reduces their systemic toxicity. Functional groups suitable for ligands, such as antibodies, peptides, nucleic acid aptamers, carbohydrates, and small molecules, further increase their specificity and efficacy [22–24].

Polymeric micelles are usually more stable in blood than liposomes and other surfactant micelles. Due to their considerably large size, these polymeric micelle systems can also be used to codeliver two or more drugs for combinational therapeutic modalities, such as radiation agents and drugs [10, 25, 26]. Polymeric micelles were recently used for the treatment of B16F10 melanoma bearing mice [27].

Paramagnetic metals, such as gadolinium (Gd) or manganese (Mn), normally used in contrast agents, can also easily be incorporated into micelles for imaging applications. Polymeric nanospheres are insoluble colloidal nano- or microparticulates possessing a polymeric core with sizes ranging from about 10 to 1000 nm. They are mostly designed as pH-sensitive drug delivery systems intended for oral delivery in order to survive in the strongly acidic environment of the stomach [28].

### 2.4. Dendrimers

Dendrimers are unimolecular, monodisperse, synthetic polymers (<15 nm) with layered architectures that constituted of a central core, an internal region consisting of repeating units and various terminal groups that determine the three-dimensional dendrimer characteristics structures. Dendrimers can be prepared for the delivery of both hydrophobic and hydrophilic drugs, nucleic acids, and imaging agents due to their attractive properties such as well-defined size and molecular weight, monodispersity, multivalency, number of available internal cavities, high degree of branching, and high number of surface functional groups [10, 28–30]. Several literature sources demonstrate the ability of dendrimer targeting ligands to induce the specific targeting and destruction of tumours. They include oligosaccharides, polysaccharides, oligopeptides, and polyunsaturated fatty acids as well as folate and tumor associated antigen [31–33]. However, a controlled release of drugs associated with dendrimers is still difficult to obtain. New developments in polymer and dendrimer chemistry have provided a new class of molecules called dendronized polymers, which are linear polymers that bear dendrons at each repeat unit, obtaining drug delivery advantages because of their enhanced circulation time. Another approach is to synthesize or conjugate the drug to the dendrimers so that incorporating a degradable link can be further used to control the release of the drug [1]. Dendrimers have also successfully been used for the therapy, immunotherapy, and radio-immunotherapy of various types of tumours [28] including melanoma [34] and squamous skin carcinoma [35]. They have also found applications in the diagnostic imaging of cancer cells, such as MRI. Gadolinium-conjugates dendrimers have allowed the selective comprehensive targeting and imaging of tumors [36].

### 2.5. Nanotubes

Carbon nanotubes belong to the family of fullerenes and are formed of coaxial graphite sheets (<100 nm) rolled up into cylinders. These structures can be obtained either as single- (one graphite sheet) or multiwalled nanotubes (several concentric graphite sheets). They exhibit excellent physical, photochemical, and electrochemical properties. Owing to their metallic or semiconductor behaviour, nanotubes are often used as biosensors. Carbon nanotubes can be also used as drug carriers and tissue-repair scaffolds [37]. Tumor targeting single-walled carbon nanotubes (SWCNT) have been synthesized by covalent attachment multiple copies of tumor-specific monoclonal antibodies, radiation ion chelates, and fluorescent probes [38]. This delivery system can be loaded with several molecules of an anticancer drug, because no covalent bonds are required, so that the increased payload does not significantly change the targeting ability of the antibody. They have also been remodelled to carry gadolinium atoms for MRI of tumors and have been surface functionalised with receptor agonists and antagonists for tumor targeting [39]. The use of carbon nanotubes in the diagnosis and treatment of melanoma has been recently reviewed [40].

### 2.6. Mesoporous Silica Nanoparticles

Mesoporous silica nanoparticles (MSN) have attracted growing interest in the last decades as an efficient drug delivery system [41–43]. Compared with conventional organic carriers, MSN have unique properties including tunable particle size and morphology, tailored mesoporous structure, uniform and tunable pore size, high chemical and mechanical stability, high surface area and pore volume, high drug-loading capacity, and easy surface functionalization [44–46].

### 2.7. Quantum Dots

Quantum dots are colloidal fluorescent semiconductor nanocrystals (2–10 nm). They possess a broad absorption band and a symmetric, narrow emission band, typically in the visible to near infrared (NIR) spectral range [47].The central core of quantum dots is usually composed of combinations of elements from groups II–VI of the periodic system (such as zinc, cadmium, selenium, and tellurium) or III–V (such as arsenic and phosphorus) [48], which are “overcoated” with a layer of ZnS. They show size- and composition-tunable emission spectra and high quantum yield. Quantum dots are photostable; therefore, the optical properties of QD make them suitable for highly sensitive, long term, and multitarget bioimaging application [49, 50]. The application to cancer detection lies in the ability to select a specific colour of light emission of QD [33]. Indeed, in order for QDs to be used for melanoma detection, the surface must be treated to increase hydrophilicity and the desired tumor-targeting ligand must be attached. Possible ligands include antibodies, peptides, and small-molecule drugs/inhibitors [51]. New approaches, such as the addition of a silica coating or a biocompatible polymer coating, have further increased the biocompatibility and reduced their toxicity. Indeed, although quantum dots offer a lot of advantages in sensing and imaging and as contrast agents in various techniques like MRI, PET, IR fluorescent imaging, and computed tomography, there is uncertainty surrounding the toxicity of the materials used.

### 2.8. Superparamagnetic Iron Oxide Nanoparticles

Superparamagnetic iron oxide nanoparticles (SPIONs) acquire a large magnetic moment in an externally applied magnetic field, thus attaining superparamagnetic behavior [49], which makes them attractive materials for advanced biomedical applications. They can be used as contrast agent in MRI [52]. They are capable of producing high contrast per unit of particles, so that small quantities of SPION are sufficient for imaging therapy, thereby reducing the toxicity issues [49, 50]. SPIONs can convert the energy supplied by an externally applied alternating magnetic field into heat [53]. This generated heat can be used for the selective destruction of tumor cells, which are more vulnerable to heating than normal body cells [49, 53]. Their surface can be engineered with a variety of functionalities, enhancing their biocompatibility and biodegradability for widespread biomedical applications [54]. In addition, polymers and capping agents can be attached to the SPION surface for increased biocompatibility and bioavailability, using biodegradable materials such as cellulose, dextran, PEG, or PLGA [54]. Recently, a prototype of carbon coated superparamagnetic iron oxide nanoparticles (SPIO@C) for sentinel lymph nodes mapping in melanoma and breast cancer patient has been developed [55].

### 2.9. Gold Nanoparticles

Gold nanoparticles (AuNP) are metallic nanoparticles. Other examples include Ag, Ni, Pt, and TiO_2_ nanoparticles. Gold nanoparticles (1–150 nm) can be prepared with different geometries, such as nanospheres, nanoshells, nanorods, or nanocages. These particles exhibit a combination of physical, chemical, optical, and electronic properties different from other biomedical nanotechnologies and provide a highly multifunctional platform for biochemical applications in the delivery of gene, imaging agents, and drugs [56, 57]. The advantages of gold nanoparticles are their ease of preparation in a range of sizes, good biocompatibility, ease of functionality, and their ability to conjugate with other biomolecules without altering their biological properties [58]. Gold nanoparticles with diameters ≤50 nm have been shown to cross the BBB [59]. They can be used to sensitize cells and tissue for treatment regimens [28], to monitor and to guide surgical procedures [60–62]. Different types of drugs, including proteins and DNA as well as smaller drug molecules, have been linked to the surface chemistry of AuNP, inducing a therapeutic effect in several types of tumors, including melanoma. They are also excellent labels for biosensors, because they can be detected by numerous techniques, such as optical absorption, fluorescence, and electric conductivity [63]. The use of the confocal reflectance microscope with antibody-conjugated AuNP has made the development of highly sensitive cancer imaging possible [64]. Furthermore, they are not toxic and biocompatible. In fact, they do not elicit any allergic or immune responses [65, 66].

## 3. Transdermal Drug Delivery Nanoparticles in Skin Cancers

**Figure 1:**
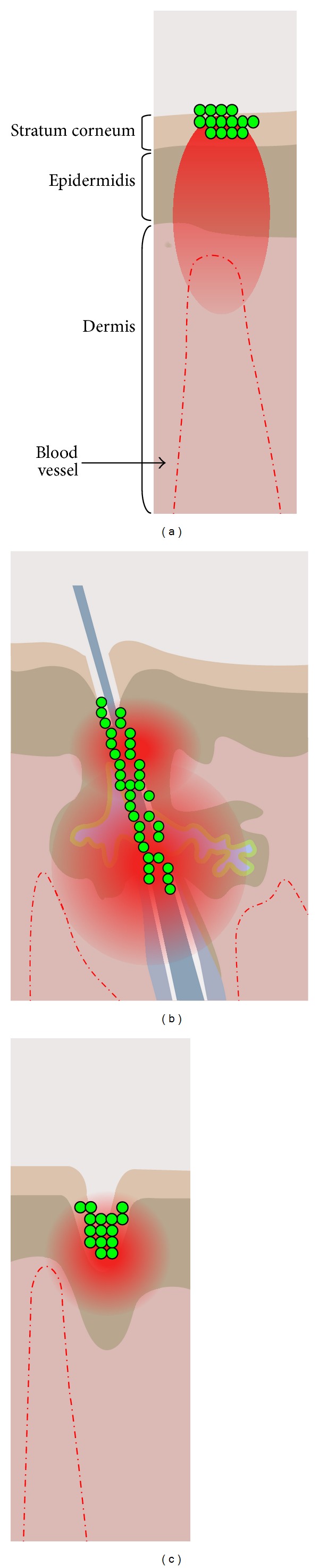
Sites in skin for nanoparticle delivery. Topical nanoparticle drug delivery takes place in three major sites: stratum corneum (SC) surface (a), openings of hair follicles (infundibulum) (b), and furrows (dermatoglyphs) (c). The nanoparticles are shown in green and the drug in red. Other sites for delivery are the viable epidermis and dermis (modified by Prow et al., [67]).

Most chemotherapeutics are administered systemically and are cytotoxic to healthy cells; therefore, cancer patients must endure considerable morbidity. The topical administration of anticancer drugs is an interesting alternative for increasing drug targeting and therapeutic benefits (Figure 1) [67]; the major challenge of this kind of treatment is to increase penetration of the antineoplastic tumor drug in sufficient levels to kill tumor cells [68]. Several techniques such as the use of chemical enhancers (i.e., oleic acid, 1-dodecylazacycloheptan-2-one or azone, dimethyl sulfoxide, propylene glycole, and ethanol) and the application of an electric field (e.g., ionophoresis, sonophoresis, and electroporation) have, therefore, been developed to successfully overcome skin barriers and to reach skin malignancies by favouring drug penetration into the deep layers of the epidermis [69]. The use of chemical penetration enhancers is the simplest strategy, causing temporary and reversible disruption of the stratum corneum and leading to increased anticancer drug penetration into the tumor. Moreover, great interest has been shown in nanoparticles delivery systems that can protect anticancer drugs against degradation and, combined with physical methods, significantly increase the tumor penetration of the drugs. Applications of nanotechnology to skin cancer has seen much effort in the design of new imaging and therapeutic approaches [70], the main focus being on diagnosing and treating metastatic melanoma. It is known that anticancer drugs showing hydrophilic properties have a low oil/water partition coefficient, high molecular weights, and ionic characters [71] and, thus, do not easily penetrate the stratum corneum. Drug permeation through the stratum corneum is regulated by Fick's second law [72]:

(1) $$\begin{array}{c} J=\frac{D_{m}C_{v}P}{L}, \end{array}$$

where *J* is the flux, *D*_*m*_ is the diffusion coefficient of the drug in the membrane, *C*_*v*_ is the drug concentration in the vehicle, *P* is the drug partition coefficient, and *L* is the stratum corneum thickness. It can be seen in the equation that the flux of a drug through the skin is governed by the diffusion coefficient of the drug in the stratum corneum, the concentration of the drug in the vehicle, the partition coefficient between the formulation and the stratum corneum, and the membrane thickness. Nanocarriers can increase drug concentration in the vehicle and so increase drug flux.

Current topical treatments for skin cancer include semisolid formulations of 5-fluorouracil [73], diclofenac [73], and imiquimod. Another topical treatment also used and approved by the US Food and Drug Administration (FDA) is photodynamic therapy (PDT) [74].

These therapies are used to treat nonmelanoma skin cancers and their precursor lesions, such as actinic keratosis. Nanocarriers could improve skin targeting, improving the drug's ability to reach and penetrate into tumor cells. Moreover, nanocarriers can improve drug stability and reduce skin irritation by avoiding direct contact of the drug with the skin's surface [75]. As indicated before, liposomes are one of the most studied nanocarriers for the treatment of cancer. They are colloidal particles composed of one or several lipid bilayers [76] biocompatible with the stratum corneum, increasing the liposome's affinity for the skin and making them able to release drugs directly to this membrane. Liposomes containing doxorubicin [77, 78], cisplatin [79, 80], oxaliplatin [81], camptothecin [82], and others have been shown to increase these drugs' cytotoxicity and to reduce side effects because of direct targeting. Some of these liposomes, such as DOXIL, are already commercially available. This liposomal formulation contains doxorubicin and was approved in the US in 1995 [78]. The topical application of anticancer drugs is, once again, primarily related to the administration of the prodrug ALA for topical PDT. Fang et al. [83] performed an in vivo study of the influence of liposomes and ethosomes in ALA skin penetration. This study showed that the flexible liposomes (ethosomes) increased 5-ALA penetration to a greater degree than did the traditional liposomes, although both formulations increased ALA penetration when compared to the control treatment. Cationic ultradeformable liposomes have also been shown to increase ALA skin permeability in vitro. In vivo, these liposomes result in persistent ALA retention in the skin and induce the production of high levels of PpIX [84]. ALA skin retention was also improved when a traditional ALA containing liposome was examined in vitro [85]. In addition to these ALA studies, 5-fluorouracil-loaded niosomes (niosomes are nonionic surfactant vesicles with a similar structure to liposomes) showed an 8-fold improvement of this drug's cytotoxicity and penetration when compared to the aqueous solution [86]. It is worth noting that liposomes in combination with other drugs not traditionally used in skin cancer treatments have also been studied. For instance, tretinoin and diclophenac-loaded liposomes [87, 88] showed improvement in these drugs' skin penetration over nonliposomal formulations. These studies were aimed at treating acne, psoriasis, and other inflammatory conditions but not skin tumors. These formulations, however, are currently proposed to treat skin cancer malignances. In summary, liposomes have been shown to increase drugs' penetration into the skin, and it appears that ultradeformable liposomes may have an even stronger effect. However, some reports describe liposome instability and drug leakage during the storage period [89].

The most investigated nanoparticles for topical delivery are solid-lipid nanoparticles and polymeric nanoparticles, such as those made from poly(dl-lactic acid) (PLA), poly(lactic-co-glycolic acid) (PLGA), and poly-*ε*-caprolactone (PCL) [90]. Both SLNs and polymeric nanoparticles have been shown to promote sustained drug release and protection against drug degradation when topically applied [91, 92]. In addition, they allow for modifications to matrix softness. It appears that nanoparticles can closely contact the superficial junctions of corneocyte clusters and furrows, possibly favoring drug accumulation for several hours. This would allow for the sustained release of anticancer drugs. However, there are controversies regarding the ideal mean diameter, flexibility, and superficial charge of nanoparticles to optimize skin penetration.

In conclusion, nanocarriers appear to be promising systems because they offer several advantages, such as low skin irritation and increased protection of encapsulated drug. An especially important advantage of these formulations is that they often increase anticancer drug penetration through the skin. The use of physical methods to improve the penetration of nanocarriers should be considered to increase the anticancer drug's penetration into the skin and to provide for targeted drug release inside tumor cells.

## 4. Drug Delivery Nanoparticles in Nonmelanoma Skin Cancers: Squamous and Basal Cell Carcinomas

Among the three main types of skin cancer: melanoma, basal cell carcinoma (BCC), and squamous cell carcinoma (SCC), BCC is the most common form, with an incidence rate that is 4 to 5 times more likely than SCC. However SCC is a common disease also, with a prevalence of more than 700,000 cases each year in the United States [93].

The risk of development of sporadic skin malignancies has been linked to ultraviolet radiation exposure, skin type, family history, prior history of skin tumors, and immunosuppression. However, a variety of hereditary syndromes can result in an increased risk of developing skin tumors, including nevoid BCC syndrome and xeroderma pigmentosum.

Excision is the gold standard treatment for localized SSC and BBC. This can be obtained through curettage and desiccation, surgical excision, radiation therapy, cryosurgery, Mohs micrographic surgery, and micrographic surgery [93].

Although the majority of SCC and BCC remain locally invasive, 1 to 5% of primary SCC may diffuse to regional lymph nodes and distant sites, such as lungs, liver, brain, and other areas of the skin [94]. On the other hand, although very rare, BCC can metastasize to distant sites of the body, which is considered a terminal condition [95].

In the case of SCC, a topical 5-fluorouracil (5-Fu) treatment is widely used when other treatments are impractical and for patients who refuse surgical treatment [96]. It is particularly useful for situations in which postoperative healing is impaired, such as lesions that involve the lower limb in elderly patients or those with venous stasis disease [97].

However, the topical application of 5-Fu often failed due to the inadequate frequency and/or length of treatment, insufficient drug concentration, and a poor penetration of 5-Fu into the epithelium, which contributes to the tumor recurrence [98].

To improve the penetration of 5-Fu and reduce many negative side effects of conventionally used chemotherapy drugs and control the release of the therapeutic agent, albumin/drug loaded magnetic nanocomposite spheres carrying 5-Fu were prepared [93]. Since albumin accumulates in tumor sites due to their altered physiology and metabolism, Misak et al. [93] demonstrated that the albumin/drug loaded magnetic nanocomposite spheres had significantly superior therapeutic effects in treating the skin cancer, with an increased efficacy to inhibit the tumor growth. The use of 5-FU-loaded polybutyl cyanoacrylate nanoparticles was carried out in local treatment of patients with basal cell carcinoma. After application of this preparation once a day for 35–40 days, 31 of 32 patients achieved histologically confirmed complete tumor resolution demonstrating that this method is preferred by patients who are not surgical candidates [99]. Photodynamic therapy (PDT) is a nonsurgical treatment that induces a cytotoxic effect by application of a photosensitizer (PS) followed by irradiation with wavelengths specific for its absorbance spectrum, in the presence of oxygen. Upon the photoirradiation of PS at specific wavelength(s), photodynamic reactions can generate cytotoxic reactive oxygen species that oxidize subcellular organelles and biomolecules, ultimately leading to the destruction of diseased cells and tissues [100]. High efficacy is demonstrated for PDT using standardized protocols in nonhyperkeratotic actinic keratoses, Bowen's disease (squamous cell carcinoma in situ), and superficial basal cell carcinomas (BCC) [101]. Two PS agents, aminolevulinic acid (ALA) and methyl aminolevulinate (MAL), are currently available for use with PDT. Aminolevulinic acid (Levulan Kerastick, DUSA Pharmaceuticals Inc., Wilmington, MA) with blue light PDT is approved for the treatment of actinic keratoses in the USA, Korea, Mexico, Brazil, Argentina, Chile, and Columbia. Methyl aminolevulinate (MAL; Metvix, Galderma, Paris, France) is licensed in Europe for PDT of actinic keratoses (AKs), Bowen's disease, and BCC [102]. Although these compounds have only been granted licenses for the treatment of actinic keratosis, the main clinical application has been in the treatment of nonmelanomatous skin lesions, mainly for basal cell carcinoma using a topical application. However, due to the hydrophilic nature of ALA, ALA-PDT has been hindered by the rate of ALA uptake into neoplastic cells and its limited penetration into tissue. A first attempt has already been performed by using liposome to better deliver ALA to the deep layers of epidermis [103]. ALA loaded nanoparticles were also prepared by using chitosan, a linear polymer composed of 2-amino-2-deoxy-*β*-D-glucan by glycosidic linkages [104]. ALA has also been carried by succinate-modified chitosan (SCHI), physically complexed with folic-acid-modified chitosan [105], to improve drug penetration and release in the cellular lysosome.

Encouraging results in the treatment of skin SCC “in vitro” have been recently obtained by Shi et al. in A431 cells, derived from human epidermoid SCC, by using poly(lactic-co-glycolic acid) (PLGA), a biomaterial developed in the 1970s and approved by the United States Food and Drug Administration (FDA), for ALA delivery [106].

Other methodological approaches to destroy SCC cells involved the use of gold nanorods, functionalized with epidermal growth factor receptor antibody conjugated with gold nanorods which have been successfully used in an “in vitro” model of human SCC, A431. Results obtained with laser photothermal therapy demonstrated that immunolabeled gold nanorods can selectively destroy the cancer cells and induce apoptosis through the ROS mediated mitochondrial pathway under low power laser exposure [107]. To prevent skin tumors induced by ultraviolet B (UVB) radiation and benzo(a)pyrene (BaP) treatment in mice, Das et al. loaded apigenin (Ap), a dietary flavonoid having an anticancer property, with poly(lactic-co-glycolide) nanoparticles (NAp) [108].

Apigenin is one of the most common dietary antioxidants, widely distributed in many fruits and vegetables and in* Lycopodium clavatum*. The topical application of apigenin in mice has been previously used to decrease the number and size of tumors in the skin induced by chemical carcinogens [109] or by UV exposure in vivo [110]. However, the nanoencapsulation of apigenin produced better effects than free apigenin, due to their smaller size and faster mobility. Moreover, NAp reduced tissue damage and showed better potential in therapeutic management of skin cancer. In the very rare cases in which local modalities are insufficient to resolve basal cell carcinoma, systemic therapy is required. No cytotoxic chemotherapy has been approved for the treatment of advanced BCC. However, with variable successes, cisplatinum-based chemotherapy regimens have been used in the past years [111]. Recent advances in the understanding of the pathogenesis of BCC have led to the development of therapeutics targeting the biological mechanism driving this malignancy. Indeed BCCs are critically dependent on a single signaling pathway, the sonic hedgehog (Shh) pathway, and the majority of BCC bearing mutations in genes in this developmental pathway [112]. Since it has been demonstrated that the inhibition of SHh-signaling can inhibit BCC tumor growth, diverse small molecule inhibitors of specific SHh signals are under study for the BCC targeted therapy [113]. However, until now, the nanoparticle-encapsulated inhibitor of the transcription factor, Gli1 (NanoHHI) belonging to the SHh pathway, has been used only in “in vitro” and “in vivo” models of human hepatic carcinoma (HCCs). In these models, Gli1 inhibition through NanoHHI has profound tumor growth inhibition and antimetastatic effects [114].

## 5. Drug Delivery Nanosystems in Melanoma

At present, the most common drug used for the treatment of melanoma is dacarbazine (DTIC), which is a US Food and Drug Administration- (FDA-) approved, first-line treatment for patients with melanomas [115].

The median survival time of patients with metastasized melanoma is only 6−10 months, and the 5-year survival rate is less than 20%. Therefore, improved treatment efficiency is urgently needed for melanoma [116–118]. As discussed above, many nanoparticles have been studied for the treatment of melanoma, including liposomes [13, 119], dendrimers, polymersomes, carbon-based nanoparticles, inorganic nanoparticles, and protein-based nanoparticles [120, 121].

It has been shown that delivering the chemotherapeutic agent doxorubicin by gold nanoparticles was very effective against a melanoma cell line [122]. Lo Prete et al. applied a cholesterol-rich nanoemulsion to deliver etoposide in a mouse model of melanoma [123]. It decreased side effects, increasing maximum tolerated dose fivefold, and increased the inhibition of tumor growth by concentrating etoposide at the tumor site (a fourfold higher concentration in tumor than with free etoposide). Doxorubicin was packed in a nanoparticle with additional antibody against CD44, to specifically target malignant cells [124]. The nanoparticle reduced the tumor size by 60% compared with untreated tumor.

In treating metastatic melanoma, solvent-based taxanes are active but demonstrate a high rate of toxicity and limited efficacy due to their water-insolubility, resulting in limited uptake and adverse reactions to the solvents used in each formulation. Using nanoparticles albumin bound paclitaxel (Nab-PTX), Hersh et al. reported a Phase II clinical trial in both previously treated and untreated melanoma patients [125] and demonstrated that nab-paclitaxel was well tolerated and active in both previously treated and chemotherapy-naive patients with metastatic melanoma. Similar results were found by Kottschade et al. in a Phase II clinical trial using nab-PTX and carboplatin in advanced melanoma, in 41 chemotherapy-naive and 35 previously treated melanoma patients. The response rate was 25.6% in the chemotherapy-naive cohort and was 8.8% in the previously treated cohort. Despite the severe side effects such as neutropenia, thrombocytopenia, neurosensory problems, fatigue, nausea, and vomiting, the authors found that the addition of bevacizumab to nab-paclitaxel and carboplatin (regimen ABC) shows promising activity in terms of both median progression-free survival and overall survival. In another clinical trial [126], vascular endothelial growth factor (VEGF) antibody increased the effect of nab-PTX [127]. Ott et al. showed that the combination of a B cell lymphoma protein (Bcl)-2 antisense oligonucleotide, temozolomide, and nab-PTX produced a response of 40.6% [128]. Similar side effects to that revealed in Kottschade et al.'s study were also reported.

From these data it, appears that, in the clinical trials, the side effects provoked by nab-PTX had, as a counterpart, a higher effectiveness against the tumor growth. In general, drug delivery nanoparticles have a higher cytotoxic effect than free drug. Indeed, it was reported that phosphatidyl-ethanolamine liposomal cisplatin had a higher cytotoxicity than classic liposomes or free cisplatin and a high level of intratumoral drug concentration for 72 h and efficiently delivered approximately 3.6 times more drug than the free drug [129]. Moreover, the anticancer therapy combining a vascular-disruptive drug (combretastatin phosphate, CA4P) and a liposomal formulation of a chemotherapeutic (doxorubicin) greatly inhibited melanoma proliferation and growth compared to monotherapies alone [130].

Liposomes containing glucocorticoids were found to be highly potent in suppressing tumor angiogenesis and inflammation at the same time [131]. Liposomal prednisolone phosphate was able to strongly inhibit endothelial cell proliferation and reduce proangiogenic protein (such as bFGF) levels, which were related to tumor angiogenesis [132].

Cationic liposome containing polyinosinic-polycytidylic acid significantly increased tyrosinase related protein (TRP)-2-specific IFN-producing cells and resulted in an augmentation of the antitumor immune response [133]. This showed another possibility in immunotherapy for melanoma by peritumoral injection. The functionalized quantum dot-liposome hybrid offered great potential for melanoma imaging due to its rapid accumulation and retention within the tumor [134, 135]. Liposomal siRNA could decrease melanoma growth and metastasis in vivo [136]. Nanotechnology has been used to deliver certain inhibitors of the MAPK pathway [137].

Given the almost universal dependence of melanomas from hyper-activation of the MAPK signalling pathway caused by activating mutation of NRAS, BRAF or loss of function mutations of the RAS-negative regulator NF1, it is of particular interest the work reported by Basu and colleagues [138], which generated and tested nanoparticles loaded with the MEK1 inhibitor PD98059 and proved its ability in enhancing the antitumor activity to cysplatinum. Such study opened a new scenario on the possibility to combine highly effective targeted-therapies in the field of melanoma such as combinations of BRAF inhibitors, MEK inhibitors, and PI3K inhibitors with the optimal delivery of the drugs also in difficult to reach sites such as brain metastasis.

Another potentially powerful application of nanoparticles involves the use of RNA interference based approaches. The possibility of tumor-selective delivery of small RNA or DNA molecules makes this application the most flexible and potentially powerful anticancer approach given that, on theory, every transcribed gene can be targeted.

A chitosan nanoparticle was used to deliver the VEGF siRNA and was demonstrated to improve the therapeutic effect. Functional graphene oxide delivered a plasmid-based STAT3 siRNA and showed significantly reduced xenografted tumor growth [139]. Indeed, STAT3 is considered to be a key mediator in melanoma which promotes brain metastasis [140]. Inhibition of phosphorylated STAT3 has been shown to increase efficacy of tumor necrosis factor- (TNF-) alpha for melanoma [141]. A nanoparticle has also been designed to carry siRNA against the oncogene* c-Myc* to target melanoma cells B16F10 and demonstrated effectiveness against melanoma [142]. Tran et al. prepared a nanoparticle to contain both siRNAs against BRAF and Akt3 which markedly increased the anticancer effect [143]. Recently, Pizzimenti et al. demonstrated that the inclusion complex of 4-hydroxynonenal, a toxic aldehyde derived from the lipid peroxidation, with a polymeric derivative of *β*-cyclodextrin enhances the antitumoral efficacy of the aldehyde in several tumor cell lines and in a three-dimensional human melanoma model [144].

Nanotechnology has been found to increase the therapeutic effect of Bcl-2 inhibition. In nude mice, oblimersen (an antisense oligonucleotide against Bcl-2) decreased xenografted melanoma growth [145]. The dual application of oblimersen with DTIC in patients in a Phase III clinical trial resulted in increased effectiveness compared with DTIC alone (9 versus 7.8 months, respectively, for overall survival; 2.6 versus 1.6 months, respectively, for progression-free survival; 13.5% versus 7.5%, respectively, for overall response; 2.8% versus 0.8%, respectively, for complete response; and 7.3% versus 3.6%, respectively, for durable response) [146]. A nanoparticle was made to carry Bcl-2 siRNA (as well as Myc and VEGF) for the treatment of melanoma [147]. It was shown that this resulted in Bcl-2 reduction in both messenger ribonucleic acid (mRNA) and protein levels. This increased the anticancer effects both in vitro and in vivo. Oblimersen has been used in a Phase I clinical trial in combination with temozolomide and nab-PTX and proved to be more effective in patients with advanced melanoma [128].

Nanoparticles have been used to deliver immunotherapy drugs, to reduce side effects [148, 149]. Yao et al. prepared a novel nanoparticle containing IL-2 and tested it in a mouse model with xenografted melanoma. The nanoparticle was made from low-molecular weight polyethylenimine (600 Da), which was linked to *β*-cyclodextrin, conjugated with folate, and further mixed with IL-2 plasmid. The new formulation inhibited tumor growth and prolonged the survival of the melanoma bearing mice [150]. Biodegradable polymer, poly(polycaprolactone), was prepared to make a nanoporous miniature device for local delivery of cytokine IFN-alpha and showed constant slow release of IFN-alpha [151]. Speiser et al. prepared a nanoparticle containing a cytosine-phosphodiester-guanine- (CpG-) loaded virus-like particle carrying melanoma antigen recognized by T cells 1 (Mart-1) to target melanoma cells, and this nanoparticle produced a strong immune response against melanoma, including increased cytotoxic CD8 T cell responses [152].

Camerin et al. used nanoparticles to carry Zn[II]-phthalocyanine disulfide (C11Pc) to test photodynamic therapy treatment efficacy in a mouse model of xenograft melanoma. It was found that a gold nanoparticle-associated C11Pc had more effective treatment outcomes. The nanoparticle had greater accumulation than did free C11Pc, and the ratio of C11Pc between melanoma and skin increased from 2.3 to 5.5 [153].

Sato et al. designed a magnetite nanoparticle by conjugating N-propionyl-cysteaminylphenol with magnetite and used this in a B16F1 xenograft mouse model. Electron microscopy demonstrated that this particle appeared only in melanoma cells. It was shown that the melanoma cells were degraded after the application of an external alternating magnetic field to increase the temperature in the tumor to 43°C. The nanoparticle had a 1.7- to 5.4-fold greater effect than the used alone magnetite did. A recent study showed that curcumin further increased the efficacy of magnetite nanoparticles [154].

In conclusion, drug delivery appears to be a promising approach for a better effective melanoma therapy.
