# Corrigendum to “Drug Delivery Nanoparticles in Skin Cancers”

**DOI:** 10.1155/2021/6158298

**Published:** 2021-02-16

**Authors:** Chiara Dianzani, Gian Paolo Zara, Giovanni Maina, Piergiorgio Pettazzoni, Stefania Pizzimenti, Federica Rossi, Casimiro Luca Gigliotti, Eric Stefano Ciamporcero, Martina Daga, Giuseppina Barrera

**Affiliations:** ^1^Department of Drug Science and Technology, University of Turin, 10125 Turin, Italy; ^2^Department of Clinical and Biological Sciences, University of Turin, 10125 Turin, Italy; ^3^Department of Genomic Medicine, University of Texas-MD Anderson Cancer Center, TX 77030 Houston, USA; ^4^Department of Public and Pediatric Health Sciences, University of Turin, 10126 Turin, Italy; ^5^Department of Health Sciences, University of Eastern Piedmont “Amedeo Avogadro”, 28100 Novara, Italy

The article titled “Drug Delivery Nanoparticles in Skin Cancers” [1] was found to contain a substantial amount of material from previously published articles [2–6]. The article has been revised as follows.

**Abstract**

Nanotechnology consists of the preparation of functional systems at the nanoscale. These systems are very attractive in nanomedicine studies and are used to apply nanotechnology in several areas of intervention such as prevention, diagnosis, and treatment of several diseases, including cancer. Over the past two decades, the rapid developments in nanotechnology have allowed the incorporation of multiple therapeutic, sensing, and targeting agents into nanoparticles, for the detection, prevention, and treatment of cancer diseases. Nanoparticles offer many advantages as a drug carrier since they can improve the solubility of poorly water-soluble drugs, improve bioavailability, modify pharmacokinetics, and increase drug half-life by reducing catabolism or the action of the immune system. They can also enable a tunable release of therapeutic compounds and the simultaneous delivery of two or more drugs for combination therapy. In this review, we discuss the recent advances in the use of diverse types of nanoparticles for systemic and topical drug delivery in the treatment of skin cancer. In particular, progress in the treatment of basal cell carcinoma, squamous cell carcinoma, and melanoma has been reported.

## 1. Introduction

Nanotechnology comprises techniques, materials, and equipment that operate at the nanoscale. It is a new approach consisting of the design, characterization, preparation, and application of structures, devices, and systems by controlling the shape and size at the nanoscale [1] . According to the federal US research and development program agency, the National Nanotechnology Initiative (NNI), nanotechnology involves the development of carrier devices or systems sized in the 1 to 100 nm range, although this limit can be extended up to 1000 nm [2] . These biomimetic features, together with their high surface-to-volume ratio and the possibility of modulating their properties, raised the interest in their use in biomedical applications with potential applications in imaging, diagnosis, and therapy [3] .

Over the past two decades, the rapid developments in nanotechnology have allowed the incorporation of multiple therapeutic, sensing, and targeting agents into nanoparticles, for the detection, prevention, and treatment of oncologic diseases.

Nanomedicine has an enormous potential to improve selectivity in targeting neoplastic cells by allowing the preferential delivery of drugs to tumors owing to the enhanced permeability and retention (EPR) effect. Furthermore, specific binding of drugs to targets in cancer cells or the tumor microenvironment increases the effectiveness of the specific treatment of cancer cells while leaving healthy cells intact. Nanoparticles (NP) can also improve the solubility of poorly water-soluble drugs, improve bioavailability, modify pharmacokinetics, and increase drug half-life by reducing catabolism or the action of the immune system. They can also enable a tunable release of therapeutic compounds and the simultaneous delivery of two or more drugs for combination therapy [4-5] . In addition, by reducing the drug doses, it is also possible to reduce side effects and ameliorate patient compliance [6] . These engineered nanocarriers also offer the opportunity to use a combination of imaging and drug therapy to monitor effects in real time, as well as the possibility of joining the delivery of drugs with energy (heat, light, and sound) for synergistic anticancer therapeutic effects [7] .

Although skin cancer is not the most deadly type of cancer, it is the most common form of cancer in the United States and many other countries [8] . Melanoma represents only a very small proportion of the skin cancer incidence, but it accounts for a major part of deaths. Indeed, at the early stage, melanoma can be surgically removed, with a survival rate of 99%, while metastasized melanoma causes the death of 80% of patients within 5 years from the diagnosis [9] . Other types of skin cancers, basal cell carcinoma and squamous cell carcinoma, are the most common diseases. Excision is the gold standard treatment for these localized diseases. However, in very rare cases, they can diffuse to regional lymph nodes and distant sites. For metastasized skin cancers, nanoparticles can represent an effective drug delivery system. Drug delivery nanoparticles can allow anticancer drugs to reach the specific cancer site and thus improve treatment efficacy. In the following sections, we illustrate the major forms of nanoparticles which have been used for systemic and transdermal drug delivery in skin cancers and the specific drug nanoparticle formulations which have been reported for the treatment of basal cell carcinoma, squamous cell carcinoma, and melanoma.

## 2. Chemicophysical Characteristics of Nanoparticles Employed for Drug Delivery in Skin Cancers

Several nanoparticles have been tested for the treatment of skin cancers, especially in melanoma treatment, including liposomes, dendrimers, polymersomes, carbon-based nanoparticles, inorganic nanoparticles, and protein-based nanoparticles. In the following paragraphs, the characteristics of the common nanoparticles used in skin cancer treatment are described.

### 2.1. Liposomes

Liposomes are phospholipid vesicles (dimensions of 50-100 nm and even larger) that have a bilayered membrane structure, similar to that of biological membranes, together with an internal aqueous phase. Liposomes are classified according to size and number of layers into multi-, oligo-, or unilamellar. The aqueous core can be used for the encapsulation of water-soluble drugs, whereas the lipid bilayers may retain hydrophobic or amphiphilic compounds. To escape from reticuloendothelial system (RES) uptake after i.v. injection, PEGylated liposomes, “stealth liposomes,” were developed for reducing clearance and prolonging circulation half-life [10] . Liposomes show excellent circulation, penetration, and diffusion properties. The possibility of linking the liposomes' surface with ligands and/or polymers increases significantly the drug delivery specificity [11] . Early research demonstrated that liposomes remain in the tumor interstitial fluid just near the tumor vessels [12] . Currently, several liposomal formulations in the clinical practice contain several drugs for the treatment of several types of cancer, including melanoma [13] . Several other liposomal chemotherapeutic drugs are at various stages of development in clinical trials. Moreover, advances with cationic liposomes led to the successful delivery of small interfering RNA (siRNA) [14] . New opportunities were proposed by Muthu and Feng [15] that developed theranostic liposomes, with the possibility of loading a wide variety of diagnostic NP along with anticancer drugs in combination with a vitamin E TPGS coating. Liposomes can also be modified to incorporate a magnetic element for use in monitoring their movement within the body using MRI [16] or to entrap gases and drugs for ultrasound-controlled drug delivery [17] .

### 2.2. Solid Lipid Nanoparticles (SLNs)

SLNs were used since the 1990s as an alternative delivery system to liposomes, emulsion, and polymeric NP. SLNs present high physical stability; i.e., they can protect the drugs against degradation, and they allow easy control of the drug release. The preparation of SLNs does not require the use of organic solvents. They are biodegradable and biocompatible and have low toxicity. In addition, the production and sterilization of SLNs on a large scale are rather easy [18] . SLNs containing docetaxel improve the efficacy of this chemotherapeutic agent in colorectal cancer cells (C-26) and malignant melanoma cells (A-375) in “in vitro” and “in vivo” experiments [19] . Cholesteryl butyrate solid lipid nanoparticles have been shown to inhibit human umbilical vein endothelial cells' adhesiveness to cancer cell lines derived from human colon-rectum, breast, and prostate cancers and melanoma [20] .

### 2.3. Polymeric Micelles and Nanospheres

Polymeric micelles are formed by two or more polymer chains with different hydrophobicity. These copolymers spontaneously assemble into a core-shell micellar structure. Specifically, hydrophobic groups form the core in order to minimize their exposure to aqueous surroundings, whereas hydrophilic groups form the corona-like shell to stabilize the core through direct contact with water [21] . The typical size of micelles for pharmaceutical applications ranges from 10 to 80 nm. Micelles, being smaller than liposomes, have a short circulation time, but they show a superior uptake by tumors because of the EPR effect. Poorly soluble drugs with high loading capacity (5–25 wt%) can be carried in the hydrophobic core, while the hydrophilic shell allows steric protection for the micelle and thereby reduce their systemic toxicity. Functional groups suitable for ligands, such as antibodies, peptides, nucleic acid aptamers, carbohydrates, and small molecules, further increase their specificity and efficacy [22-24] .

Polymeric micelles are usually more stable in blood than liposomes and other surfactant micelles. Due to their considerably large size, these polymeric micelle systems can also be used to codeliver two or more drugs for combinational therapeutic modalities, such as radiation agents and drugs [10, 25, 26] . Polymeric micelles were recently used for the treatment of B16F10 melanoma-bearing mice [27] . Paramagnetic metals, such as gadolinium (Gd) or manganese (Mn), normally used as contrast agents, can also be incorporated into micelles for imaging applications. Polymeric nanospheres are insoluble colloidal nano- or microparticles having a polymeric core with sizes ranging from about 10 to 1000 nm. They are mostly designed as pH-sensitive drug delivery systems intended for oral delivery in order to survive in the strongly acidic environment of the stomach [28] .

### 2.4. Dendrimers

Dendrimers are unimolecular, monodispersed, synthetic polymers (<15 nm) with layered architectures consisting of a central core, an internal region of repeating units, and various terminal groups that determine the three-dimensional dendrimer characteristic structures. Dendrimers can be prepared for the delivery of hydrophobic and hydrophilic drugs, nucleic acids, and imaging agents. These properties are due to their special features, such as well-defined size and molecular weight, monodispersity, multivalency, the number of available internal cavities, a high degree of branching, and a high number of surface functional groups [10, 28-30] . Several literature sources demonstrate the ability of dendrimer-targeting ligands to induce specific targeting and reduction of the tumor mass. They include oligosaccharides, polysaccharides, oligopeptides, and polyunsaturated fatty acids, as well as folate and tumor-associated antigens [31-33] . However, a controlled release of drugs carried by dendrimers is still difficult to obtain. New research in polymer and dendrimer chemistry has developed a new class of molecules called dendronized polymers, which are linear polymers with dendrons at each repeating unit that have drug delivery advantages because of their enhanced circulation time. Another approach is to synthesize or conjugate the drug to the dendrimers so that incorporating a degradable link allows further control of the release of the drug [1] . Dendrimers have also been successfully used for the therapy, immunotherapy, and radioimmunotherapy treatment of various types of cancers [28] , including melanoma [34] and squamous skin carcinoma [35] . They have also been used in the diagnostic imaging of cancer cells, such as MRI. Gadolinium-conjugate dendrimers have also allowed the selective, comprehensive targeting and imaging of tumors [36] .

### 2.5. Nanotubes

Carbon nanotubes are a type of fullerenes and are formed of coaxial graphite sheets (<100 nm) rolled up into cylinders. They can be obtained as either single- (one graphite sheet) or multiwalled nanotubes (several concentric graphite sheets). They exhibit excellent physical, photochemical, and electrochemical properties. Owing to their metallic or semiconductor behavior, nanotubes are often used as biosensors. Carbon nanotubes can also be used as drug carriers and tissue repair scaffolds [37] . Tumor-targeting single-walled carbon nanotubes (SWCNT) can be obtained by covalent attachment of multiple copies of tumor-specific monoclonal antibodies, radiation ion chelates, and fluorescent probes [38] . This delivery system can be used to load several molecules of anticancer drugs because no covalent bonds are required so that the increased load does not significantly change the targeting ability of the antibody. They have also been remodeled to carry gadolinium atoms for MRI of tumors and have been surface-functionalized with receptor agonists and antagonists for tumor targeting [39] . The use of carbon nanotubes in the diagnosis and treatment of melanoma has been recently reviewed [40] .

### 2.6. Mesoporous Silica Nanoparticles

Mesoporous silica nanoparticles (MSN) have attracted growing interest in the last few decades as an efficient drug delivery system [41-43]. Compared with conventional organic carriers, MSN have unique properties, including tunable particle size and morphology, tailored mesoporous structure, uniform and tunable pore size, high chemical and mechanical stability, high surface area and pore volume, high drug loading capacity, and easy surface functionalization [44-46] .

### 2.7. Quantum Dots

Quantum dots are colloidal fluorescent semiconductor nanocrystals (2-10 nm). They possess a broad absorption band and a symmetric, narrow emission band, typically in the visible to near-infrared (NIR) spectral range [47]. The central core of quantum dots is usually composed of combinations of elements from groups II-VI of the periodic table (such as zinc, cadmium, selenium, and tellurium) or III-V (such as arsenic and phosphorus) [48] , which are “overcoated” with a layer of ZnS. They show size- and composition-tunable emission spectra and high quantum yield. Quantum dots are photostable; therefore, the optical properties of QD make them suitable for highly sensitive, long-term, and multitarget bioimaging applications [49, 50] . The application to cancer detection lies in the ability to select a specific color of light emission of QD [33] . Indeed, in QDs used for melanoma detection, the surface must be treated to increase hydrophilicity and covered with the specific tumor-targeting ligands. Possible ligands include antibodies, peptides, and small-molecule drugs/inhibitors [51]. New approaches, such as the addition of a silica coating or a biocompatible polymer coating, have further increased the biocompatibility and reduced their toxicity. Indeed, although quantum dots offer a lot of advantages in sensing and imaging and as contrast agents in various techniques like MRI, PET, IR fluorescent imaging, and computed tomography, there is uncertainty surrounding the toxicity of the materials used.

### 2.8. Superparamagnetic Iron Oxide Nanoparticles

Superparamagnetic iron oxide nanoparticles (SPIONs) acquire a magnetic moment when applied to an external magnetic field, thus attaining superparamagnetic behavior [49 ]. These characteristics make SPIONs an attractive tool for advanced biomedical applications. They can be used as a contrast agent in MRI [52] . SPIONs can produce high contrast per unit of particles. This reduces the toxicity of these particles because a small amount of SPION is enough for imaging therapy [49, 50] . Moreover, SPIONs can convert into heat the energy supplied by an externally applied alternating magnetic field [53] . The heat, generated by SPION, can be used for the selective destruction of tumor cells, which are more vulnerable to heating than normal cells [49, 53] . Their surface can be functionalized by the attachment of polymers and capping agents, using biodegradable materials such as cellulose, dextran, PEG, or PLGA, enhancing their biocompatibility and biodegradability for several biomedical applications [54]. Recently, a prototype of carbon-coated superparamagnetic iron oxide nanoparticles (SPIO@C) for sentinel lymph node mapping in melanoma and breast cancer patients has been developed [55, 56] .

### 2.9. Gold Nanoparticles

Gold nanoparticles (AuNP) are metallic nanoparticles. Other examples include Ag, Ni, Pt, and TiO_2_ nanoparticles. Gold nanoparticles (1-150 nm) can be prepared with different geometries, such as nanospheres, nanoshells, nanorods, or nanocages. These particles exhibit a combination of physical, chemical, optical, and electronic properties different from other nanoparticles and provide a highly multifunctional platform for biochemical applications in the delivery of nucleic acids, imaging agents, and drugs [57, 58] . The gold nanoparticles are easily prepared in a range of sizes, have good biocompatibility, are easily functionalized, and can be conjugated with other biomolecules without altering their biological properties [59]. Gold nanoparticles with diameters ≤50 nm can cross the BBB [60]. They can be used to sensitize cells and tissue for treatment regimens [28] , to monitor, and to guide surgical procedures [61, 62] . Different types of drugs, including proteins and DNA, as well as smaller drug molecules, have been linked to the surface chemistry of AuNP, inducing a therapeutic effect in several types of tumors, including melanoma. They are also excellent labels for biosensors because they can be detected by numerous techniques, such as optical absorption, fluorescence, and electric conductivity [63] . The use of the confocal reflectance microscope with antibody-conjugated AuNP has made possible the development of highly sensitive imaging in cancer application [64, 65] . Furthermore, they are not toxic and biocompatible. In fact, they do not elicit any allergic or immune responses [66] .

## 3. Transdermal Drug Delivery Nanoparticles in Skin Cancers

The systematic administration of most chemotherapeutic drugs causes serious toxic effects in cancer patients; thus, their topical administration could reduce the general toxicity (Figure 1) [67] . Obviously, the topical treatment must be performed so that the drug reaches the deepest skin layers affected by the tumor in concentrations able to kill tumor cells [68] . Several techniques, such as the use of chemical enhancers (i.e., oleic acid, azone, dimethyl sulfoxide, propylene glycol, and ethanol) and the application of an electric field (e.g., ionophoresis, sonophoresis, and electroporation), have been developed to increase drug penetration into the deep layers of the skin and reach the tumor mass [69] . Chemical enhancers provoke disruption of the stratum corneum, which is reversible. For this reason, the increase of anticancer drug penetration into the tumor is temporary. In this contest, the studies about the use of nanoparticles for transdermal drug delivery have gained great interest. Nanoparticles can shield anticancer drugs from degradation and significantly increase penetration of the drugs into the tumor mass. Applications of nanotechnology to skin cancer have seen much effort in the design of new imaging and therapeutic approaches [70] , with the main focus being on diagnosing and treating metastatic melanoma. Anticancer drugs with hydrophilic properties show characteristics such as a low oil/water partition coefficient, high molecular weights, and electric potential that do not allow them to reach the stratum corneum [71, 72] . Drug permeation through the stratum corneum is regulated by Fick's second law [73] :

(1) $$\begin{array}{c} J=\frac{\text{Dm}∙\text{Cv}∙P}{L}. \end{array}$$

In this formula, *J* represents the flux, Dm represents the diffusion coefficient of the drug in the membrane, Cv represents the drug concentration in the vehicle, *P* represents the drug partition coefficient, and *L* represents the thickness of the stratum corneum. In the equation, the flux of a drug through the skin is governed by the diffusion coefficient of the drug in the stratum corneum, the concentration of the drug in the vehicle, the partition coefficient between the formulation and the stratum corneum, and the membrane thickness. The drug concentration can be increased in nanoparticles, thus enhancing the drug flux.

In nonmelanoma skin cancers, the current topical treatments are composed of semisolid formulations of 5-fluorouracil, diclofenac [74] , and imiquimod. Moreover, photodynamic therapy (PDT) is another topical treatment approved by the US Food and Drug Administration (FDA) [75] . The use of nanoparticles to carry the anticancer drugs could improve the penetration and the retention of the drug into tumor cells and could reduce skin irritation by avoiding direct contact of the drug with the skin's surface [76] . As indicated before, liposomes, particles composed of one or several lipid bilayers, are one of the most studied nanocarriers for the treatment of cancer [77] . They are biocompatible with the stratum corneum and are able to release drugs directly to this layer. It has been demonstrated that liposomes loaded with doxorubicin [78, 79] , cisplatin [80, 81] , oxaliplatin [82] , camptothecin [83] , and others increased cytotoxicity of these drugs and reduced side effects. Some of these liposomes, such as Doxil®, have been approved by the FDA in 1995 [79] . The application of anticancer drugs to the skin can be accompanied by the treatment with the prodrug ALA for topical PDT. Fang et al. [84] demonstrated that the flexible liposomes (ethosomes) increased 5-ALA penetration to a greater extent than traditional liposomes, although both formulations increased ALA penetration when compared to the control treatment.

Oh et al. demonstrated that topical delivery of 5-ALA loaded in cationic ultradeformable liposomes increased ALA skin permeability “in vitro” [85] . In “in vivo” kinetic studies performed by Pierre et al., the authors demonstrated an increase in skin ALA accumulation when formulations containing 10% oleic acid were used; ALA skin retention was also increased when examined in vitro [86] .

In addition to these ALA studies, 5-fluorouracil-loaded niosomes (niosomes are nonionic surfactant vesicles with a similar structure to liposomes) showed an 8-fold increase of drug cytotoxicity and penetration with respect to the aqueous solution [87] .

Other studies have been performed to test the skin penetration of drugs used to treat acne, psoriasis, and other inflammatory conditions but not skin cancer. It has been demonstrated that liposomes loaded with tretinoin and diclofenac [88, 89] increase skin penetration with respect to the free drug. Although liposomes have been shown to increase drug penetration into the skin, in particular ultradeformable liposomes, some reports describe liposome instability and drug leakage during the storage period [90] .

Solid lipid nanoparticles (SLNs) and polymeric nanoparticles, such as those made from poly(dl-lactic acid) (PLA), poly(lactic-co-glycolic acid) (PLGA), and poly-*ε*-caprolactone (PCL), are the most investigated nanoparticles for topical delivery [91]. It has been demonstrated that both SLNs and polymeric nanoparticles enhance permeation and improve skin stability and drug release [92, 93] . It appears that nanoparticles can adhere to the superficial junctions of corneocyte clusters so that they can promote drug accumulation and release for a long time.

In conclusion, nanoparticle-based formulation approaches appear to be promising systems because they minimize peripheral exposure and maximize tumor accumulation. In particular, in skin cancer treatments, they can increase anticancer drug penetration. Consistent with the increased penetration, the use of physical methods also increases the penetration of nanocarriers into the skin, suggesting that they can further augment the application of nanoparticle-based delivery in skin cancer treatments.

## 4. Drug Delivery Nanoparticles in Nonmelanoma Skin Cancers: Squamous and Basal Cell Carcinomas

Among the three main types of skin cancer: melanoma, basal cell carcinoma (BCC), and squamous cell carcinoma (SCC), BCC is the most common form, with an incidence rate that is 4 to 5 times more likely than SCC. However, SCC is a common disease also, with a prevalence of more than 700,000 cases each year in the United States [94] .

The risk of development of sporadic skin malignancies has been linked to ultraviolet radiation exposure, skin type, family history, prior history of skin tumors, and immunosuppression. However, a variety of hereditary syndromes can result in an increased risk of developing skin tumors, including nevoid BCC syndrome and xeroderma pigmentosum.

Excision is the gold standard treatment for localized SCC and BCC. This can be obtained through curettage and desiccation, surgical excision, radiation therapy, cryosurgery, Mohs micrographic surgery, and micrographic surgery [94] .

Although the majority of SCC and BCC remain locally invasive, 1 to 5% of primary SCC may diffuse to regional lymph nodes and distant sites, such as the lungs, liver, brain, and other areas of the skin [95]. On the other hand, although very rare, BCC can metastasize to distant sites of the body, which is considered a terminal condition [96] .

In the case of SCC, a topical 5-fluorouracil (5-Fu) treatment is widely used when other treatments are impractical and for patients who refuse surgical treatment [97] . It is particularly useful for situations in which postoperative healing is impaired, such as lesions that involve the lower limb in elderly patients or those with venous stasis disease [98] .

However, the topical application of 5-Fu often failed due to the inadequate frequency and/or length of treatment, insufficient drug concentration, and poor penetration of 5-Fu into the epithelium, which contributes to the tumor recurrence [99] . To improve the penetration of 5-Fu, reduce many side effects of chemotherapy drugs, and control the release of the therapeutic agent, albumin/drug-loaded magnetic nanocomposite spheres carrying 5-Fu were prepared [100] . Since albumin accumulates in tumor sites due to their altered physiology and metabolism, Misak et al. [100] demonstrated that the albumin/drug-loaded magnetic nanocomposite spheres had significantly superior therapeutic effects in treating skin cancer, with increased efficacy to inhibit the tumor growth. The use of 5-Fu-loaded poly (butyl cyanoacrylate) nanoparticles was carried out in the local treatment of patients with basal cell carcinoma. After application of this preparation once a day for 35-40 days, 31 of 32 patients achieved histologically confirmed complete tumor resolution demonstrating that this method is preferred by patients who are not surgical candidates [101] . Photodynamic therapy (PDT) is a nonsurgical treatment that induces a cytotoxic effect by application of a photosensitizer (PS) followed by irradiation with wavelengths specific for it in the presence of oxygen. The photoirradiation of PS at specific wavelengths generates cytotoxic reactive oxygen species (ROS) able to oxidize subcellular organelles and biomolecules, ultimately leading to the killing of cancer cells [102] . High efficacy is demonstrated for PDT using standardized protocols in nonhyperkeratotic actinic keratoses, Bowen's disease (SCC in situ), and superficial BCC [103] . Two PS agents, aminolevulinic acid (ALA) and methyl aminolevulinate (MAL), are currently available for use with PDT. ALA (Levulan Kerastick®, DUSA Pharmaceuticals Inc., Wilmington, MA) with blue light PDT is approved for the treatment of actinic keratoses in the US, Korea, Mexico, Brazil, Argentina, Chile, and Columbia. MAL (Metvix®, Galderma, Paris, France) is licensed in Europe for PDT of actinic keratoses, Bowen's disease, and BCC [104]. Although these compounds have only been granted licenses for the treatment of actinic keratosis, the main clinical application has been in the treatment of nonmelanomatous skin lesions, mainly for BCC using a topical application. However, due to the hydrophilic nature of ALA, ALA-PDT has been hindered by the rate of ALA uptake into neoplastic cells and its limited penetration into tissue. A first attempt has already been performed by using liposomes to better deliver ALA to the deep layers of the epidermis [105]. ALA-loaded nanoparticles were also prepared by using chitosan, a linear polymer composed of 2-amino-2-deoxy-*β*-D-glucan by glycosidic linkages [106] . ALA has also been carried by succinate-modified chitosan (SCHI), physically complexed with folic acid-modified chitosan [107] , to improve drug penetration and release in the cellular lysosome.

Encouraging results in the treatment of skin SCC “in vitro” have been recently obtained by Shi et al. in A431 cells, derived from human epidermoid SCC, by using poly(lactic-co-glycolic acid) (PLGA), a biomaterial developed in the 1970s and approved by the United States FDA, for ALA delivery [108] .

Other methodological approaches to destroy SCC cells involved the use of gold nanorods, functionalized with the epidermal growth factor receptor antibody conjugated with gold nanorods, which have been successfully used in an “in vitro” model of human SCC, A431. Results obtained with laser photothermal therapy demonstrated that immunolabeled gold nanorods can selectively destroy the cancer cells and induce apoptosis through the ROS-mediated mitochondrial pathway under low-power laser exposure [109] . To prevent skin tumors induced by ultraviolet B (UVB) radiation and Benzo(a)pyrene (BaP) treatment in mice, Das et al. loaded apigenin (Ap), a dietary flavonoid having an anticancer property, with poly(lactic-co-glycolide) nanoparticles (NAp) [110] .

Apigenin is one of the most common dietary antioxidants, widely distributed in many fruits and vegetables and in *Lycopodium clavatum*. The topical application of apigenin in mice has been previously used to decrease the number and size of tumors in the skin induced by chemical carcinogens [111] or by UV exposure in vivo [112] . However, the nanoencapsulation of apigenin produced better effects than free apigenin due to their smaller size and faster mobility. Moreover, NAp reduced tissue damage and showed better potential in the therapeutic management of skin cancer.

In the very rare cases in which local modalities are insufficient to resolve basal cell carcinoma, systemic therapy is required. No cytotoxic chemotherapy has been approved for the treatment of advanced BCC. However, with variable success, cisplatinum-based chemotherapy regimens have been used in past years [113] . Recent advances in the understanding of the pathogenesis of BCC have led to the development of therapeutics targeting the biological mechanism driving this malignancy. Indeed, BCCs are critically dependent on a single signaling pathway, the Sonic hedgehog (Shh) pathway, and the majority of BCC-bearing mutations in genes in this developmental pathway [114] . Since it has been demonstrated that the inhibition of Shh signaling can inhibit BCC tumor growth, diverse small-molecule inhibitors of specific Shh signals are under study for the BCC targeted therapy [115] . However, until now, the nanoparticle-encapsulated inhibitor of the transcription factor, Gli1 (NanoHHI) belonging to the Shh pathway, has been used only in “in vitro” and “in vivo” models of human hepatic carcinoma (HCC). In these models, Gli1 inhibition through NanoHHI has profound tumor growth inhibition and antimetastatic effects [116] .

## 5. Drug Delivery Nanosystems in Melanoma

At present, therapeutic approaches for melanoma include surgical resection, chemotherapy, PDT, immunotherapy, biochemotherapy, and targeted therapy.

The most common conventional drug, employed for the treatment of disseminated melanoma, is dacarbazine (DTIC), which is a United States FDA-approved first-line treatment, although it does not seem to improve the overall survival (OS) of patients [117] .

Since the median survival time of patients with metastasized melanoma is only 6–10 months, and the 5-year survival rate is less than 20%, the identification of new therapeutic approaches for melanoma treatment is an urgent and unmet need [118-120] . As discussed above, several nanoparticles have been studied for the treatment of melanoma, including liposomes [13,121] , dendrimers, polymersomes, carbon-based nanoparticles, inorganic nanoparticles, and protein-based nanoparticles [122-123] .

It has been shown that delivering doxorubicin by gold nanoparticles strongly affects the growth of melanoma cells [124] . Lo Prete et al. studied the effect of etoposide carried by a cholesterol-rich nanoemulsion (LDE) in a mouse model of melanoma [125] . These authors demonstrated that etoposide-LDE retains the cytotoxicity of the etoposide in free form and drastically reduced the drug toxicity since the maximum tolerated dose was approximately fivefold greater than in commercial etoposide. Moreover, this etoposide formulation was concentrated fourfold in the tumor compared with the normal adjacent tissues.

In a study aimed at evaluating whether CD44 targeting was a way to selectively deliver therapeutic agents encapsulated inside colloidal delivery systems, doxorubicin was encapsulated inside colloidal delivery systems linked to antibodies against CD44 [126] . CD44-targeted liposomes reduced the tumor size in melanoma-bearing mice to 60% of that in the untreated control, whereas nontargeted liposomes were ineffective.

In animal models, solvent-based taxanes are active in treating tumors. Their high rate of toxicity and their limited efficacy can be attributed to their water insolubility, resulting in limited uptake and adverse reactions to the solvents used in each formulation. Since in previous studies, nanoparticle albumin-bound Paclitaxel (nab-Paclitaxel) demonstrated a better antitumor activity and a higher intratumoral concentration, Hersh et al. investigated its use in a phase II clinical trial in patients with metastatic melanoma. These authors demonstrated that nab-Paclitaxel was well tolerated and active in both the previously treated and chemotherapy-naive patients [127] . Similar results were found by Kottschade et al. [128] . In a phase II clinical trial using nab-PTX and carboplatin in advanced melanoma, 41 chemotherapy-naive and 35 previously treated melanoma patients demonstrated that this treatment increased median progression-free survival and overall survival. In another clinical trial conducted in chemotherapy-naive patients with unresectable stage IV melanoma, the addition of bevacizumab to nab-Paclitaxel and carboplatin increased the anticancer activity despite tolerability issues [129] . In a phase I trial performed by enrolling chemotherapy-naive patients with metastatic melanoma and normal LDH levels, Ott et al. demonstrated that the combination of oblimersen, a Bcl-2 antisense oligonucleotide, and nab-Paclitaxel produced a response rate of 40.6% [130].

From these data, it appears that in the clinical trials, the side effects provoked by nab-PTX had, as a counterpart, higher effectiveness against tumor growth. In general, drug delivery nanoparticles have a higher cytotoxic effect than the free drug. Indeed, it was reported that phosphatidylethanolamine liposomal cisplatin had higher cytotoxicity than classic liposomes or free cisplatin and a high level of intratumoral drug concentration for 72 h and efficiently delivered approximately 3.6 times more drugs than the free drug [131] . Moreover, the anticancer therapy combining a vascular-disruptive drug (combretastatin phosphate, CA4P) and a liposomal formulation of a chemotherapeutic (doxorubicin) greatly inhibited melanoma proliferation and growth compared to monotherapies alone [132] .

Glucocorticoids encapsulated in long-circulating liposomes were found to have strong cytotoxic effects on B16F10 melanoma cells, and the anticancer effect was sustained by the antiproliferative action towards angiogenic endothelial cells [133] . These results are in agreement with previous data demonstrating that liposomal prednisolone phosphate was able to inhibit cancer cell proliferation by reduction of the intratumoral production of the majority of proangiogenic factors. These effects are the result of prolonged high levels of prednisolone in the tumor by liposomal delivery [134] .

Cationic liposome containing polyinosinic-polycytidylic acid significantly increased tyrosinase-related protein- (TRP-) 2-specific IFN-producing cells and resulted in an augmentation of the antitumor immune response [135] . The stimulation of innate immune response was also obtained by peritumoral injection of a complex composed of cationic liposomes and polyinosinic-polycytidylic acid [136] or functionalized-quantum dot-liposome hybrids [137] .

It has been reported that small interfering RNA (siRNA) incorporated into neutral liposomal decreased melanoma growth and metastasis in vivo [138] . Moreover, Inamdar et al. reported, in a comprehensive review, the diverse possibilities of inhibiting certain components of the MAPK pathway through the use of nanotechnologies [139] .

Given the almost universal dependence of melanomas from hyperactivation of the MAPK signaling pathway caused by activating the mutation of NRAS and BRAF or loss of function mutations of the RAS-negative regulator NF1, Basu and colleagues [140] , generated and tested nanoparticles loaded with the MEK1 inhibitor PD98059 and proved its ability to enhance the antitumor activity against cisplatinum. Such studies opened a new possibility of combining highly effective targeted therapies in the field of melanoma, such as combinations of BRAF inhibitors, MEK inhibitors, and PI3K inhibitors with the optimal delivery of the drugs also in difficult-to-reach sites such as brain metastasis.

Another potentially powerful application of nanoparticles involves the use of RNA interference-based approaches. The possibility of tumor-selective delivery of small RNA or DNA molecules makes this application the most flexible and potentially powerful anticancer approach given that, in theory, every transcribed gene can be targeted.

Since STAT3 is considered a key mediator in melanoma progression that is able to promote brain metastasis [141] , Yin et al. investigated whether graphene oxide, chemically functionalized with polyethylenimine and polyethylene glycol, could function as a plasmid-based STAT3-specific siRNA carrier in mouse malignant melanoma. Results obtained showed significant regression in tumor growth and tumor weight after the treatment [142] . In addition to a key role in cell proliferation, STAT3 has been identified as a mediator of immune suppression in melanoma patients. Combinational therapy of STAT3 blockade agents with IFN-alpha enhanced both the innate and adaptive cytotoxic T-cell activities in a syngeneic intracerebral murine tumor model of melanoma [143] . Chen et al. described different nanoparticles designed to carry siRNA against the well-known oncogene *c*-*Myc* in the B16F10 melanoma cell line [144] . Nanoparticles composed of N,N-distearyl-N-methyl-N-2-(N′-arginyl) aminoethyl ammonium chloride, a guanidinium-containing cationic lipid, showed a strong effect in reducing tumor growth and in sensitizing the melanoma cells to Paclitaxel [143] . Nanoliposomal-mediated siRNA targeting of ^V600E^B-Raf and Akt3 acting cooperatively resulted in an approximately 65% decrease in early or invasive cutaneous melanoma compared with inhibition of each singly with negligible associated systemic toxicity. A nanoliposomal ultrasound-mediated approach has been developed by Tran et al. for delivering siRNA targeting of ^V600E^B-Raf and Akt3 into both melanocytic tumors present in the skin or melanoma cell lines. These authors found that this approach inhibited early lesion development and prevented cutaneous melanoma metastasis [145] . Recently, Pizzimenti et al. demonstrated that the inclusion complex of 4-hydroxynonenal, a toxic aldehyde derived from the lipid peroxidation, with a polymeric derivative of *β*-cyclodextrin enhances the antitumoral efficacy of the aldehyde in several tumor cell lines and in a three-dimensional human melanoma model [146] .

The expression of the antiapoptotic gene Bcl-2 is increased in several tumors, including some melanomas. Thus, its inhibition can increase the tumor cell susceptibility to apoptosis. Benimetskaya et al. reported that oblimersen (an antisense oligonucleotide against Bcl-2) decreased xenografted melanoma growth [147] . The same substance in combination with dacarbazine significantly improved multiple clinical outcomes in patients with advanced melanoma and increased overall survival [148] . The arginine-grafted bioreducible poly(disulfide amine) polymer (ABP) was used to carry a siRNA cocktail targeting Bcl-2, Myc, and VEGF. Beloor et al. suggested that two administrations of this formulation could regress advanced-stage tumors, including melanoma [149] . The combined treatment with oblimersen, temozolomide, and nab-Paclitaxel was well tolerated and improved the response in patients with advanced melanoma [150] .

Antigen delivery through nanoparticles has been developing as a vaccine strategy. Biodegradable poly(D,L-lactide-co-glycolide) nanoparticles (PLGA-NP) carrying murine melanoma antigenic peptides and mannosylated nanoparticles loaded with messenger RNA (mRNA), which enhance the transfection of dendritic cells (DCs), have been used, with encouraging results, for vaccination of melanoma-bearing mice [151, 152] . Another strategy to stimulate the immunological response in a xenograft mouse model of melanoma was investigated by Yao et al., which prepared polyethylenimine linked by *β*-cyclodextrin conjugated with folate nanoparticles carrying IL-2. Results demonstrated that this formulation had high effectiveness and could represent an alternative gene therapy strategy for melanoma [153]. It has been reported that a local administration of cytokines can have beneficial effects in locally recurrent melanoma. By using a biodegradable polymer for local delivery of the IFN-alpha, which permitted a slow release of cytokines, He et al. obtained a remarkable antitumor effect in a human xenograft model of melanoma [154] . The stimulation of immune response in a phase I/II study enrolling stage II-IV melanoma patients was obtained by using virus-like nanoparticles loaded with a ligand for toll-like receptor linked to the melanoma-specific Melan-A/MART-1 peptide. These nanoparticles produced a CD8 T-cell response and conferred long-term immune protection from the disease [155] .

To test the effectiveness of nanoparticles carrying Zn[II]-phthalocyanine disulfide (C11Pc) in the photodynamic therapy (PDT) of a subcutaneously transplanted amelanotic melanoma, Camerin et al. intravenously injected phthalocyanine either in free form or bound to gold nanoparticles in C57 mice bearing amelanotic melanoma. It was found that C11Pc-loaded nanoparticles had a greater accumulation in melanoma cells, with respect to surrounding tissues, than did free C11Pc, and PDT studies showed a markedly more significant antitumor response in mice receiving the nanoparticle-bound photosensitizer [156] .

For intracellular hyperthermia treatment of melanoma cells, Sato et al. prepared nanoparticles by conjugating N-propionyl-cysteaminylphenol with magnetite. When cells treated with NPrCAP/M are heated to 43°C, melanoma cells were degraded more significantly than by magnetite alone. The efficacy of this formulation was confirmed in reducing the growth of transplanted B16 melanoma [157] .

In conclusion, drug delivery by nanoparticles appears to be a promising approach for better effective melanoma therapy.

**References**

[1] R. Singh and J.W. Jr Lillard, “Nanoparticle-based targeted drug delivery,” Experimental and Molecular Pathology, vol. 86, no. 3, pp. 215-223, 2009.

[2] D.J. Bharali, M. Khalil, M. Gurbuz, T.M. Simone and S.A. Mousa, “Nanoparticles and cancer therapy: a concise review with emphasis on dendrimers,” International Journal of Nanomedicine. Vol. 4, no. 1, pp.1-7, 2009.

[3] N. Sanvicens and M.P. Marco, “Multifunctional nanoparticles-properties and prospects for their use in human medicine,” Trends in Biotechnology, vol. 26, no.8, pp. 425-433, 2008.

[4]T. M. Allen and P.R. Cullis, “Drug delivery systems: entering the mainstream,” Science, vol. 303. no. 5665, pp. 1818-1822, 2004.

[5]D.F: Emerich and C.G. “Thanos, The pinpoint promise of nanoparticle-based drug delivery and molecular diagnosis,” Biomolecular Engineering, vol. 23, no. 4, pp. 171-184, 2006.

[6]R.K. Jain and T. Stylianopoulos, “Delivering nanomedicine to solid tumors,” Nature Reviews Clinical Oncology, vol. 7, no. 11, pp. 653-664, 2010.

[7]L.S. Jabr-Milane, L.E. van Vlerken, S. Yadav and M.M. Amiji, “Multi-functional nanocarriers to overcome tumor drug resistance,” Cancer Treatment Reviews, vol. 34, no. 7, pp. 592-602, 2008.

[8]H. Misaka, N. Zachariasb, Z. Songc, S. Hwangd, K.P. Mana, R. Asmatulua and S.Y. Yangb, “Skin cancer treatment by albumin/5-Fu loaded magnetic nanocompositespheres in a mouse model,” Journal of Biotechnology, vol. 164, no.1, pp. 130-136, 2013.

[9]A.F. Jerant, J.T. Johnson, C.D. Sheridan and T.J. Caffrey, “Early detection and treatment of skin cancer,” Am Fam Physician, vol. 62, no. 2, pp. 357–368, 2000.

[10]L. Zhang, and N. Zhang, “How nanotechnology can enhance docetaxel therapy,” International Journal of Nanomedicine, vol.8, no.1, pp. 2927-2941, 2013.

[11] V.P. Torchilin, “Recent advances with liposomes as pharmaceutical carriers,” Nature Reviews Drug Discovery, vol. 4, no. 2, pp.145-160, 2005.

[12] F. Yuan, M. Leunig, S.K. Huang, D.A. Berk, D. Papahadjopoulos and R.K. Jain, “Microvascular permeability and interstitial penetration of sterically stabilized (stealth) liposomes in a human tumor xenograft,” Cancer Reserch, vol. 54, no 1, pp. 3352-3356, 1994.

[13]M. Slingerland, H.J. Guchelaar and H. Gelderblom, “Liposomal drug formulations in cancer therapy: 15 years along the road,” Drug Discovery Today, vol. 17. No. 3-4, pp. 160-166, 2012.

[14] J. Yano, K. Hirabayashi, S. Nakagawa, T. Yamaguchi, M. Nogawa, I. Kashimori, H. Naito, H. Kitagawa, K. Ishiyama, T. Ohgi and T. Irimura, “Antitumor activity of small interfering RNA/cationic liposome complex in mouse models of cancer,” Clinical Cancer Research, vol. 10, no. 22, pp. 7721-7726, 2004.

[15] M.S. Muthu and S.S. Feng, “Theranostic liposomes for cancer diagnosis and treatment: current development and pre-clinical success,” Expert Opinion on Drug Delivery, vol. 10, no. 2, pp. 151-155, 2013

[16] S.J.H. Soenen, J. Cocquyt, L. Defour, P. Saveyn, P. Van Der Meeren and M. De Cuyper, Design and development of magnetoliposome-based Theranostics, Materials and Manufacturing Processes, vol. 23, no. 6, pp. 611-614, 2008).

[17] S.L. Huang, “Liposomes in ultrasonic drug and gene delivery,” Advanced Drug Delivery Reviews, vol. 60, no.1, pp. 1167–1176, 2008.

[18] W. Mehnert and K. Mäder, “Solid lipid nanoparticles: production, characterization and applications,” Advanced Drug Delivery Reviews, vol. 47. No. 2-3, pp. 165-196, 2001.

[19] N. Mosallaei, M.R. Jaafari, M.Y. Hanafi-Bojd, S. Golmohammadzadeh and B. Malaekeh-Nikouei, “Docetaxel-loaded solid lipid nanoparticles: preparation, characterization, in vitro, and in vivo evaluations,” Journal of Pharmaceutical Sciences, vol. 102, no. 6, pp. 1994-2004, 2013

[20] R. Minelli, L. Serpe, P. Pettazzoni, V. Minero, G. Barrera, C. Gigliotti, R, Mesturini, A.C. Rosa, P. Gasco P, N. Vivenza, E. Muntoni, R. Fantozzi, U. Dianzani, G.P. Zara and C. Dianzani C, “Cholesteryl butyrate solid lipid nanoparticles inhibit the adhesion and migration of colon cancer cells,” British Journal of Pharmacology, vol. 166, no. 2, pp. 587-601, 2012.

[21] V.P. Torchilin, “Micellar nanocarriers: pharmaceutical perspectives,” Pharmaceutical Research, vol. 24, no. 1, pp. 1-16, 2007

[22] M.J. Fonseca, J.C. Jagtenberg, H.J. Haisma and G. Storm, “Liposome-mediated targeting of enzymes to cancer cells for site-specific activation of prodrugs: comparison with the corresponding antibody-enzyme conjugate,” Pharmaceutical Research, vol. 20, no. 3, pp. 423- 428, 2003.

[23] A. Schnyder, S. Krähenbühl, J. Drewe and J. Huwyler, “Targeting of daunomycin using biotinylated immunoliposomes: pharmacokinetics, tissue distribution and in vitro pharmacological effects,” Journal of Drug Targeting, 2005 Jun;13(5):325-35.

[24] O.C. Farokhzad, J. Cheng, B.A. Teply, I. Sherifi, S. Jon, P.W Kantoff, J.P. Richie and R. Langer, “Targeted nanoparticle-aptamer bioconjugates for cancer chemotherapy in vivo,” Proceedings of the National Academy of Sciences, vol. 103, no. 16, pp.6315-6320, 2006.

[25] L. Zhang, A.f. Radovic-Moreno, F. Alexis, F.X. Gu, P.A. Basto, V. Bagalkot, S. Jon, R.S. Langer and O.C. Farokhzad, “Co-delivery of hydrophobic and hydrophilic drugs from nanoparticle-aptamer bioconjugates,” ChemMedChem, vol. 2, no. 9, pp. 1268-1271, 2007.

[26] L. Zhang, F.X. Gu,J.M. Chan, A.Z. Wang, R.S. Langer and O.C. Farokhzad, “Nanoparticles in medicine: therapeutic applications and developments,” Clinical Pharmacology & Therapeutics, Vol. 83, no. 5, pp.761-769, 2008.

[27] M. Coimbra, C.J. Rijcken, M. Stigter, W.E. Hennink, G. Storm and R.M. Schiffelers, “Antitumor efficacy of dexamethasone-loaded core-crosslinked polymeric micelles,” Journal of Controlled Release, vol. 163, no. 3, pp. 361-7, 2012.

[28] S. Nazir, T. Hussain, A. Ayub, U. Rashid and A.J. Macrobert, “Nanomaterials in combating cancer: Therapeutic applications and developments,” Nanomedicine. 2013 Jul 19. pii: S1549-9634(13)00339-0. doi: 10.1016/j.nano.2013.07.001. [Epub ahead of print]

[29] C.M. Paleos, D. Tsiourvas, Z. Sideratou and L.A.Tziveleka, “Drug delivery using multifunctional dendrimers and hyperbranched polymers,” Expert Opinion on Drug Delivery, vol. 7, no. 12, pp. 1387-1398, 2010.

[30] S. Svenson, “Dendrimers as versatile platform in drug delivery applications,” European Journal of Pharmaceutics and Biopharmaceutics, vol. 2, no. 1, pp. 445-462, 2009.

[31] S. Jaracz, J. Chen, L.V. Kuznetsova and I. Ojima, “Recent advances in tumor-targeting anticancer drug conjugates,” Bioorganic & Medicinal Chemistry, vol.|13, no. 17, pp. 5043-5054, 2005.

[32] C. Kojima, K. Kono, K. Maruyama and T. Takagishi, “Synthesis of polyamidoamine dendrimers having poly(ethylene glycol) grafts and their ability to encapsulate anticancer drugs,” Bioconjugate Chemistry, vol. 11, no. 6, pp. 910-917, 2000.

[33] J. LaRocque, D.J. Bharali and S.A. Mousa, “Cancer detection and treatment: the role of nanomedicines,” Molecular Biotechnology, vol. 42, no. 3, pp 358-366 2009.

[34] J. Chen, R. Shao, X.D. Zhang and C. Chen, “Applications of nanotechnology for melanoma treatment, diagnosis, and theranostics,” International Journal of Nanomedicine, vol. 8, no. 1, pp. 2677-2688, 2013.

[35] S. Battah, S. Balaratnam, A. Casas, S. O'Neill, C. Edwards, A. Batlle, P. Dobbin P and A.J. MacRobert, “Macromolecular delivery of 5-aminolaevulinic acid for photodynamic therapy using dendrimer conjugates,” Molecular Cancer Therapeutics, vol. 6, no. 3, pp. 876-885, 2007.

[36] H. Kobayashi H and M.W. Brechbiel, “Dendrimer-based macromolecular MRI contrast agents: characteristics and application,” Molecular Imaging, vol.2, no. 1, pp. 1-10, 2003,

[37] S. Polizu, O. Savadogo, P. Poulin and L. Yahia, “Applications of carbon nanotubes-based biomaterials in biomedical nanotechnology,” Journal of Nanoscience and Nanotechnology, vol. 6, no. 7, pp. 1883-1904, 2006.

[38] M.R. McDevitt, D. Chattopadhyay, B.J. Kappel, J.S. Jaggi, S.R. Schiffman, C. Antczak, J.T. Njardarson, R. Brentjens and D.A. Scheinberg, “Tumor Targeting with Antibody-Functionalized, Radiolabeled Carbon Nanotubes,” The journal of nuclear medicine,vol. 48, no.7, 1180-1189, 2007.

[39] S. Bosi, T. Da Ros, G. Spalluto and M. Prato, “Fullerene derivatives: an attractive tool for biological applications,” European Journal of Medicinal Chemistry, vol.38, no. 11-12, pp. 913-923, 2003.

[40] N. Naderi, S.Y. Madani, E. Ferguson, A. Mosahebi and A.M. Seifalian, “Carbon nanotubes in the diagnosis and treatment of malignant melanoma,” Anticancer Agents in Medicinal Chemistry, vol. 13, no. 1, pp. 171-185, 2013.

[41] I.I. Slowing, B.G. Trewyn, S. Giri and V.S.Y. Lin. “Mesoporous Silica Nanoparticles for Drug Delivery and Biosensing Applications,”Advanced Functional Materials, vol. 17, no. 1, 1225–1236, 2007.

[42] I.I. Slowing, J.L. Vivero-Escoto, C.W. Wu and V.S.Y. Lin, “Mesoporous silica nanoparticles as controlled release drug delivery and gene transfection carriers,” Advanced Drug Delivery Reviews, vol. 60, no. 1, pp. 1278–1288, 2008.

[43] F. Torney, B.G. Trewyn, V.S.Y. Lin and K. Wang, “Mesoporous silica nanoparticles deliver DNA and chemicals into plants,” Nature Nanotechnology, vol. 2, no. 1, pp. 295 – 300, 2007.

[44] I. Slowing, B.G. Trewyn and V.S.Y. Lin, “Effect of Surface Functionalization of MCM-41-Type Mesoporous Silica,” Journal of the American Chemical Society, vol. 128, no.1, pp. 14792-14793, 2006.

[45] V. Cauda, A. Schlossbauer, J. KechtA. Zürner A and T. Bein, “Multiple core-shell functionalized colloidal mesoporous silica nanoparticles,” Journal of the American Chemical Society, vol. 131, no. 32, pp. 11361-11370, 2009.

[46] C.P. Tsai, C.Y. Chen, Y. Hung, F. H. Chang and C.Y. Mou, “Monoclonal antibody functionalized mesoporous silica nanoparticles (MSN) for selective targeting breast cancer cells,” Journal of Material Chemistry, vol.19, no. 1, pp. 5737–5743, 2009.

[47] N.M. Idris, M.K. Gnanasammandhan, J. Zhang, P.C. Ho, R. Mahendran and Y. Zhang, “In vivo photodynamic therapy using upconversion nanoparticles as remote-controlled nanotransducers,” Nature Medicine, vol. 18, no. 10, pp. 1580-1585, 2012.

[48] Y. Wang Y and L. Chen, “Quantum dots, lighting up the research and development of nanomedicine,” Nanomedicine, vol. 7, no. 4, pp. 385-402, 2011.

[49] A.P. Alivisatos, “Semiconductor clusters, nanocrystals, and quantum dots,” Science, vol. 271, no. 1, pp. 933-937, 1996.

[50] H.C. Huang, S. Barua, G. Sharma, S.K. Dey and K. Rege, “Inorganic nanoparticles for cancer imaging and therapy,” Journal of Controlled Release, vol. 155, no. 3, pp. 344-357, 2011.

[51] I.L. Medintz, H.T. Uyeda, E.R. Goldman and H. Mattoussi, “Quantum dot bioconjugates for imaging, labelling and sensing,” Nature Materials, vol. 4, no. 6, pp. 435-446, 2005.

[52]E. Ruoslahti, S.N. Bhatia and M.J. Sailor, “Targeting of drugs and nanoparticles to tumors,” The Journal of Cell Biology, vol. 188, no. 6, pp. 759-68, 2010.

[53]J. T, Y. Zhao, Y. Ding anb G. Nie, “Using functional nanomaterials to target and regulate the tumor microenvironment: diagnostic and therapeutic applications,” Advanced Materials, vol. 25, no. 1, pp. 3508-3525, 2013.

[54]M. Johannsen, U. Gneveckow, K. Taymoorian, B. Thiesen, N. Waldöfner, R. Scholz, K. Jung, A. Jordan, P. Wust and S.A. Loening, “Morbidity and quality of life during thermotherapy using magnetic nanoparticles in locally recurrent prostate cancer: results of a prospective phase I trial,” International Journal of Hyperthermia. Vol. 23, no. 3, pp. 315-323, 2007.

[55]F.M. Kievit and M. Zhang, “Surface engineering of iron oxide nanoparticles for targeted cancer therapy,” Accounts of Chemical Research, vol. 44, no. 10, pp. 853-862, 2011.

[56]Y.X. Wang, D.W. Wang, X.M. Zhu, F. Zhao and K.C. Leung, “Carbon coated superparamagnetic iron oxide nanoparticles for sentinel lymph nodes mapping,” Quantitative Imaging in Medicine and Surgery, vol. 2, no. 1, pp. 53-56, 2012.

[57]C.L. Zavaleta, B.R. Smith, I. Walton, W. Doering, G. Davis, B. Shojaei, M.J. Natan https://www.pnas.org/content/106/32/13511.short-aff-2 and S.S. Gambhir, “Multiplexed imaging of surface enhanced Ramanscattering nanotags in living mice using noninvasive Raman spectroscopy,”Proceedings of the National Academy of Sciences of the United States of America, vol. 106, no. 32, pp. 13511–13516, 2009.

[58]W. Lu, Q. Huang, G. Ku, X. Wen, M. Zhou, D. Guzatov, P. Brecht, R. Su, A. Oraevsky, L.V. Wang and C. Li,“Photoacoustic imaging of living mouse brain vasculature using hollow gold nanospheres,” Biomaterials, vol. 31, no 9, pp. 2617–2626, 2010.

[59]R. Albrecht, In J.E. Beesley (Ed.), Immunocytochemistry: a practical approach, Vol 2, Oxford University Press, Oxford, 1993, Chapter 7).

[60]G. Sonavane, K. Tomoda and K. Makino, “Biodistribution of colloidal gold nanoparticles after intravenous administration: Effect of particle size,” Colloids and Surfaces B: Biointerfaces vol. 66, no. 1, pp. 274-280, 2008.

[61]E.C. Dreaden, L.A. Austin, M.A. Mackey and M.A. El-Sayed, “Size matters: gold nanoparticles in targeted cancer drug delivery,” Therapeutic Delivery vol. 3, no. 4, pp. 457-478, 2012.

[62] X.M. Qian, X.H. Peng, D.O. Ansari, Q. Yin-Goen, G. Z Chen, D.M. Shin, L. Yang, A.N. Young, M.D. Wang and Shuming Nie, “In vivo tumor targeting and spectroscopic detection with surface-enhanced raman nanoparticle tags,” Nature Biotechnology, vol. 26, no.1, 83-90, 2008.

[63]J.V. Jokerst and S.S. Gambhir, “Molecular imaging with theranostic nanoparticles,” Accounts of Chemical Research, vol. 44, no. 10, pp. 1050–1060, 2011.

[64]X. Huang, P.K. Jain, I.H. El-Sayed and M.A. El-Sayed, “Gold nanoparticles: interesting optical properties and recent applications in cancer diagnostics and therapy,” Nanomedicine (Lond). Vol. 2, no. 5, pp. 681-693, 2007.

[65]J. Kimling, M. Maier, B. Okenve, V. Kotaidis, H. Ballot and A. Plech, “Turkevich method for gold nanoparticle synthesis revisited,” The Journal of Physical Chemistry B, vol. 110, no. 32, pp. 15700–15707, 2006.

[66]J.F. Hainfeld, F.A. Dilmanian, D.N. Slatkin and H.M. Smilowitz, “Radiotherapy enhancement with gold nanoparticles,” Journal of Pharmacy and Pharmacology, vol. 60, no. 8, pp. 977–985, 2008.

[67]Y. Pan, S. Neuss, A. Leifert, M. Fischler,F. Wen, U. Simon, G. Schmid, W.Brandau and W. Jahnen-Dechent, “Size-dependent cytotoxicity of gold nanoparticles. Small, vol. 3, no, 11, pp. 1941–1949, 2007.

[68]T. W. Prow, J. E. Grice, L. L. Lin, Lin LL, R. Faye, M. Butler, W. Becker, E.M. Wurm, C. Yoong, T.A. Robertson,H.P. Soyer, and M.S. Roberts, “Nanoparticles and microparticles for skin drug delivery”, Advanced Drug Delivery Reviews, vol 63, no. 6, pp. 470-491, 2011.

[69]L. A. DeLouise, “Applications of Nanotechnology in Dermatology,” Journal of investigative dermatology, vol. 132, no. 3, pp. 964-975, 2012.

[70]S. F. Taveira and R. F. Lopez, “ Topical administration of anticancer drugs for skin cancer treatment,” In Skin Cancers Risk Factors, Prevention and Therapy”, Caterina La Porta (Ed.), pp 247-272, 2011.

[71]M.B. Weiss, E. Andrew, A.E. Aplin, “Paying particle attention to novel melanoma treatment strategies,” Journal of Investigation Dermatology, vol.130, pp.2699–2701, 2010.

J. G. Souza, G. M. Gelfuso, P. S. Simão et al., “Iontophoretic transport of zinc phthalocyanine tetrasulfonic acid as a tool to improve drug topical delivery,” Anticancer Drugs, vol. 22, no. 8, pp. 783-93, 2011.

[72]J. G. Souza, G. M. Gelfuso, P. S. Simão et al., “Iontophoretic transport of zinc phthalocyanine tetrasulfonic acid as a tool to improve drug topical delivery,” Anticancer Drugs, vol. 22, no. 8, pp. 783-93, 2011.

[73]A.C. Williams, B.W. Barry, “ “Penetration enhancers,” Advanced Drug Delivery Reviews, vol.56, no.5, pp. 603-618, 2004.

[74]M. V. Barrera, E. Herrera, “ Topical chemotherapy of actinic keratosis and non-melanoma skin cancer: current options and future perspectives,” Actas Dermo- Sifiliográficas, vol.98, no.8, pp. 556-562, 2007.

[75]E. M. Galiczynski, A. T. Vidimos, “ Nonsurgical treatment of nonmelanoma skin cancer,” Dermatologic Clinics,” vol.29, pp. 297-309, 2011.

[76] M. H. Schmid, H. C. Korting, “Therapeutic progress with topical liposome drugs for skin disease,” Advanced Drug Delivery Reviews, vol.18, no.3, pp.335-342, 1996.

[77]T. Gratieri, G. M. Gelfuso, R. F. V. Lopez, and E. B. Souto, “ Current efforts and the potential of nanomedicine in treating fungal keratitis,” Expert Review of Ophthalmology, vol.5, no.3, pp. 365-384, 2010.

[78]J. Hosoda, S. Unezaki, K. Maruyama, et al., “ Antitumor activity of doxorubicin encapsulated in poly(ethylene glycol)-coated liposomes,” Biological & Pharmaceutical Bulletin, vol.18, no.9, pp. 1234-1237, 1995.

[79]Y. Barenholz, “ Liposome application: problems and prospects,” Current Opinion in Colloid & Interface Science, vol.6, no.1, pp. 66-77, 2001.

[80]D. Lasic, “ Structure and Structure-Activity Relationships of Lipid-Based Gene Delivery Systems,” In: Leaf Huang, Mien-Chie Hung and Ernst Wagner, Editor(s), Nonviral Vectors for Gene Therapy, Academic Press, San Diego, pp. 69-89, 1999.

[81]M. L. Krieger, N. Eckstein, V. Schneider, et al., “Overcoming cisplatin resistance of ovarian cancer cells by targeted liposomes in vitro,” International Journal of Pharmaceutics, vol.389, no.1-2, pp. 10-17, 2010.

[82]A. S. Lila, Y. Doi, K. Nakamura, et al., “ Sequential administration with oxaliplatincontaining PEG-coated cationic liposomes promotes a significant delivery of subsequent dose into murine solid tumor,” Journal of Controlled Release, vol.142, no.2, pp. 167-173, 2010.

[83]M. Watanabe, K. Kawano, K. Toma, et al., “ In vivo antitumor activity of camptothecin incorporated in liposomes formulated with an artificial lipid and human serum albumin,” Journal of Controlled Release, vol.127, no.3, pp. 231-238, 2008.

[84]Y. P. Fang, Y. H. Tsai, Y. B. Huang, “ Comparison of 5-aminolevulinic acid-encapsulated liposome versus ethosome for skin delivery for photodynamic therapy,” International Journal of Pharmaceutics, vol.356, no.1-2, pp. 144- 152, 2008.

[85]E. K. Oh, S. E. Jin, J. K. Kim, et al., “ Retained topical delivery of 5-aminolevulinic acid using cationic ultradeformable liposomes for photodynamic therapy,” European Journal of Pharmaceutical Sciences, vol 44, no. 1-2, pp. 149-157, 2011.

[86]M. B. Pierre, E. Jr. Ticci, A. C. Tedesco, and M. V. Bentley, “ Oleic acid as optimizer of the skin delivery of 5-aminolevulinic acid in photodynamic therapy,” Pharmaceutical Research, vol.23, no.2, pp. 360-366, 2006.

[87]D. Paolino, D. Cosco, R. Muzzalupo, et al., “ Innovative bola-surfactant niosomes as topical delivery systems of 5-fluorouracil for the treatment of skin cancer,” International Journal of Pharmaceutics, vol.353, no.1-2, pp. 233-242, 2008.

[88]S. Kitagawa, M. Kasamaki, “ Enhanced delivery of retinoic acid to skin by cationic liposomes,” Chemical & Pharmaceutical Bulletin, vol. 54, no.2, pp. 242-244, 2006.

[89]G. M. E. Zaafarany, G. A. S. Awad, S. M. Holyel, and N. D. Mortada, “ Role of edge activators and surface charge in developing ultradeformable vesicles with enhanced skin delivery,” International Journal of Pharmaceutics, vol.397, no.1-2, pp.164-172, 2010.

[90]M. Glavas-Dodov, E. Fredo-kumbaradzi, K. Goracinova, et al., “The effects of lyophilization on the stability of liposomes containing 5-FU,” International Journal of Pharmaceutics, vol. 291, no. 1-2, pp. 79-86, 2005.

[91]F. Rancan, D. Papakostas, S. Hadam, et al., “Investigation of polylactic acid (PLA) nanoparticles as drug delivery systems for local dermatotherapy,” Pharmaceutical Research, vol.26, no.8, pp. 2027-2036, 2009.

[92]Z. Teixeira, B. Zanchetta, B. Melo, et al., “Retinyl palmitate flexible polymeric nanocapsules: characterization and permeation studies,” Colloids and Surfaces B: Biointerfaces, vol.81, no.1, pp. 374-380, 2010.

[93]F. Marquele-Oliveira, D. Santana, S.F. Taveira, et al., “Development of nitrosyl ruthenium complexloaded lipid carriers for topical administration: improvement in skin stability and in nitric oxide release by visible light irradiation,”. Journal of Pharmaceutical and Biomedical Analysis, vol.53, no.4, pp. 843-851, 2010.

[94]H. Misaka, N. Zachariasb, Z. Songc, S. Hwangd, K.-P. Mana, R. Asmatulua, and S.-Y. Yang, “Skin cancer treatment by albumin/5-Fu loaded magnetic nanocomposite spheres in a mouse model”, Journal of Biotechnology, vol. 164, no.1, pp. 130– 136, 2013.

[95]M. G. Joseph, W. P. Zulueta, and P. J. Kennedy, “Squamous cell carcinoma of the skin of the trunk and limbs: the incidence of metastases and their outcome”, The Australian and New Zealand Journal of Surgery, vol. 62, no. 9, pp. 697–701, 1992.

[96]A. Wadhera, M. Fazio, G. Bricca, and O. Stanton, “Metastatic basal cell carcinoma: a case report and literature review. How accurate is our incidence data?”, Dermatology Online Journal, vol. 12, no. 5, pp. 7, 2006.

[97]H. M. Sturm, “Bowen's disease and 5-fluorouracil”, Journal of the American Academy of Dermatology, vol. 1, no. 6, pp. 513–522, 1979.

[98]I. Mandekou-Lefaki, F. Delli, T. Koussidou-Eremondi, O. Mourellou-Tsatsou, and A. Dionyssopoulos A, “Imiquimod 5% cream: a new treatment for Bowen's disease”, International Journal of Tissue Reaction, vol. 27, no.1, pp. 31-8, 2005.

[99]D. K. Goette, “Topical chemotherapy with 5-fluorouracil”, Journal of the American Academy of Dermatology, vol. 4, no.6, pp. 633-49, 1981.

[100]H. Misak, N. Zacharias, Z. Song, S. Hwang, K.-P. Mana, R. Asmatulua, and S.-Y. Yang. “Skin cancer treatment by albumin/ loaded magnetic nanocomposite spheres in a mouse model”, Journal of Biotechnology, vol. 164, no.1, pp.130– 136, 2013.

[101] M. Hadjikirova, P. Troyanova, and M. Simeonova, “Nanoparticles as drug carrier system of 5-fluorouracil in local treatment of patients with superficial basal cell carcinoma”, Journal of BUON, vol. 10, no. 4, pp. 517-21, 2005.

[102]Z. Huang, H. Xu, A. D. Meyers, A.I. Musani, L. Wang, R. Tagg, A. B. Barqawi, and Y. K Chen, “Photodynamic therapy for treatment of solid tumors – potential and technical challenges”, Technology in Cancer Research and Treatment, vol. 7, no.4, pp. 309-320, 2008.

[103]C. A. Morton, R.-M. Szeimies, A. Sidoroff, and L. R. Braathen, “European guidelines for topical photodynamic therapy part 1: treatment delivery and current indications – actinic keratoses, Bowen's disease, basal cell carcinoma”, Journal of the European Academy of Dermatology and Venereology, vol. 27, no.5, pp. 536–544, 2013.

[104]C. Morton, K.E. McKenna, and L. E. Rhodes, “Guidelines for topical photodynamic therapy: update”, British Journal of Dermatology, vol. 159, no. 6, pp. 1245–1266, 2008.

[105]A. Casas, and A. Batlle, “Aminolevulinic acid derivatives and liposome delivery as strategies for improving 5-aminolevulinic acid-mediated photodynamic therapy”, Current Medicinal Chemistry, vol. 13, no.10, pp. 1157–68, 2006.

[106]S. J. Yang, M. J. Shieh, F. H. Lin, P. J. Lou, C. L. Peng, M. F. Wei, C. J. Yao, P. S. Lai, and T. H. Young, “Colorectal cancer cell detection by 5-aminolaevulinic acid-loaded chitosan nanoparticles”, Cancer Letters, vol. 273, no. 2, pp. 210–220, 2009.

[107]S. J. Yang, C. F. Lin, M. L. Kuo, and C. T. Tan, “Photodynamic detection of oral cancers with high-performance chitosan-based nanoparticles Biomacromolecules”, Biomacromolecules, vol. 14, no. 9, pp. 3183-3191, 2013.

[108]L. Shi, X. Wang, F. Zhao, H. Luan, Q. Tu, Z. Huang, H. Wang, and H. Wang, “In vitro evaluation of 5-aminolevulinic acid (ALA) loaded PLGA nanoparticles”, International Journal of Nanomedicine, vol. 8, pp. 2669–2676, 2013.

[109]C. S. Rejiya, Jatish Kumar, V. Raji, M. Vibin, and A. Abraham “Laser immunotherapy with gold nanorods causes selective killing of tumour cells”, Pharmacological Research, vol. 65 no.2, pp. 261– 269, 2012.

[110]S. Das, J. Das, A. Samadder, A. Paul, and A. R. Khuda-Bukhsh, “Efficacy of PLGA-loaded apigenin nanoparticles in Benzo[a]pyrene and ultraviolet-B induced skin cancer of mice: Mitochondria mediated apoptotic signalling cascades”, Food and Chemical Toxicology, vol. 62, pp. 670–680, 2013.

[111] H. Wei, L. Tye, E. Bresnick, and D. F. Birt, “Inhibitory effect of apigenin, a plant flavonoid, on epidermal ornithine decarboxylase and skin tumor promotion in mice”, Cancer Research, vol. 50, no. 3, pp. 499-502, 1990.

[112]D. F. Birt, D. Mitchell, B. Gold, P. Pour, and H. C. Pinch, “Inhibition of ultraviolet light induced skin carcinogenesis in SKH-1 mice by apigenin, a plant flavonoid”, Anticancer Research, vol.17, no. 1A, pp. 85-91, 1997.

[113]P. Pfeiffer, O. Hansen, and C. Rose, “Systemic cytotoxic therapy of basal cell carcinoma. A review of the literature”, European Journal of Cancer, vol. 26, no. 1, pp. 73-77, 1990.

[114]H. Hahn, C. Wicking, P.G. Zaphiropoulous, A. Chidambaram, B. Gerrard, I. Vorechovsky, A.E. Bale, R. Toftgard, M. Dean, and B. Wainwright, “A mammalian patched homolog is expressed in target tissues of sonic hedgehog and maps to a region associated with developmental abnormalities”, The Journal of Biological Chemistry, vol. 271, no. 21, pp. 12125-12128, 1996.

[115]J. K. Iwasaki, D. Srivastava, R. L.Moy, H. J. Lin, and D. J. Kouba, “The molecular genetics underlying basal cell carcinoma pathogenesis and links to targeted therapeutics”. Journal of The American Academy of Dermatology, vol. 66, no. 5, pp. e167-178, 2012.

[116]Y. Xu, V. Chenna, C. Hu, H. X. Sun, M. Khan, H. Bai, X.R. Yang, Q. F. Zhu, Y. F. Sun, A. Maitra, J. Fan, and R. A. Anders, “Polymeric nanoparticle-encapsulated hedgehog pathway inhibitor HPI-1 (NanoHHI) inhibits systemic metastases in an orthotopic model of human hepatocellular carcinoma”, Clinical Cancer Research, vol. 18, no. 5, pp. 1291-1302, 2012.

[117]T. K. Eigentler, U. M. Caroli, P. Radny, and C. Garbe. “Palliative therapy of disseminated malignant melanoma: a systematic review of 41 randomised clinical trials”, Lancet Oncology, vol. 4, no. 12, pp. 748–759, 2003.

[118]C. I. Falkson, J. Ibrahim, J. M. Kirkwood, A. S. Coates, M. B. Atkins, and R. H. Blum, “Phase III trial of dacarbazine versus dacarbazine with interferon alpha-2b versus dacarbazine with tamoxifen versus dacarbazine with interferon alpha-2b and tamoxifen in patients with metastatic malignant melanoma: an Eastern Cooperative Oncology Group study”, Journal of Clinical Oncology, vol. 16, no. 5, pp. 1743–1751, 1998.

[119]M. B. Atkins, J. Hsu, S. Lee, G. I. Cohen, L. E. Flaherty, J. A. Sosman, V. K. Sondak, J. M. Kirkwood, and Eastern Cooperative Oncology Group, “Phase III trial comparing concurrent biochemotherapy with cisplatin, vinblastine, dacarbazine, interleukin-2, and interferon alfa-2b with cisplatin, vinblastine, and dacarbazine alone in patients with metastatic malignant melanoma (E3695): a trial coordinated by the Eastern Cooperative Oncology Group”. Journal of Clinical Oncology, vol. 26, no. 35, pp. 5748–5754, 2008.

[120]A. Sharma, A. K. Sharma, S. V. Madhunapantula, D. Desai, S. J. Huh, P. Mosca, S. Amin, and G. P. Robertson, “Targeting Akt3 signaling in malignant melanoma using isoselenocyanates” Clinical Cancer Research, vol. 15, no. 5, pp. 1674–1685, 2009.

[121]M. A. Tran, R. J. Watts, and G. P. Robertson, “Use of liposomes as drug delivery vehicles for treatment of melanoma”, Pigment Cell and Melanoma Research, vol. 22, no. 4, 2009

[122]D. Bei, J. Meng, and B. B. Youan, “Engineering nanomedicines for improved melanoma therapy: progress and promises”, Nanomedicine (Lond), vol. 5, no. 9, pp. 1385–1399, 2010.

[123]I. Pacheco, C. Buzea, and V. Tron, “Towards new therapeutic approaches for malignant melanoma”, Expert Reviews in Molecular Medicine, vol.13, e33, 2011.

[124]X. Zhang, H. Chibli, D. Kong, and J. Nadeau, “Comparative cytotoxicity of golddoxorubicin and InP-doxorubicin conjugates”, Nanotechnology. 2012;23(27):275103.

[125]A. C. Lo Prete, D. A. Maria, D. G. Rodrigues, C. J. Valduga, O. C. Ibañez, and R. C. Maranhão “Evaluation in melanoma-bearing mice of an etoposide derivative associated to a cholesterol-rich nano-emulsion”, Journal of Pharmacy and Pharmacology, vol. 58, no. 6, pp. 801–808, 2006.

[126]M. W. Ndinguri, A. Zheleznyak, J. L. Lauer, C. J. Anderson, and G.B. Fields, “Application of collagen-model triple-helical peptide-amphiphiles for CD44-Targeted drug delivery systems”, Journal of Drug Delivery, vol. 2012, pp. 592602, 2012.

[127]E. M. Hersh, S. J. O'Day, A. Ribas, W. E. Samlowski, M. S. Gordon, D. E. Shechter, A. A. Clawson, and R. Gonzalez “A phase 2 clinical trial of nab-paclitaxel in previously treated and chemotherapy-naive patients with metastatic melanoma”, Cancer, vol. 116, no. 1, 155–163, 2010.

[128]L. A. Kottschade, V. J. Suman, T. Amatruda 3rd, R. R. McWilliams, B. I. Mattar, D. A. Nikcevich, R. Behrens, T. R. Fitch, A. J. Jaslowski, and S. N. Markovic, “A phase II trial of nab-paclitaxel (ABI-007) and carboplatin in patients with unresectable stage IV melanoma: a North Central Cancer Treatment Group Study, N057E(1)”, Cancer, vol. 117, no. 8, pp. 1704– 1710, 2011.

[129]L. A. Kottschade, V. J. Suman, D. G. Perez, R. R. McWilliams, J. S. Kaur, T. T. Amatruda 3rd, F. J. Geoffroy, H. M. Gross, P. A. Cohen, A. J. Jaslowski, M. L. Kosel, and S.N. Markovic, “A randomized phase 2 study of temozolomide and bevacizumab or nab-paclitaxel, carboplatin, and bevacizumab in patients with unresectable stage IV melanoma: a North Central Cancer Treatment Group study, N0775”, Cancer, vol. 119, no. 3, pp. 586–592, 2013.

[130]P. A. Ott, J. Chang, K. Madden, R. Kannan, C. Muren, C. Escano, X. Cheng, Y. Shao, S. Mendoza, A. Gandhi, L. Liebes, and A. C. Pavlick, “Oblimersen in combination with temozo¬lomide and albumin-bound paclitaxel in patients with advanced melanoma: a phase I trial”, Cancer Chemotherapy and Pharmacology, vol. 71, no. 1, pp. 183–191, 2013.

[131]T. L. Hwang, W. R. Lee, S. C. Hua, and J. Y. Fang, “Cisplatin encapsulated in phosphatidylethanolamine liposomes enhances the in vitro, cytotoxicity and in vivo, intratumor drug accumulation against melanomas”, Journal of Dermatological Science, vol. 46, no. 11–20, 2007.

[132]I. Mitrus, A. Sochanik, T. Cichon, and S. Szala, “Combination of combretastatin A4 phosphate and doxorubicin-containing liposomes affects growth of B16–F10 tumors”, Acta Biochimica Polonica, vol. 56, pp. 161-165, 2009.

[133]M. Banciu, J. M. Metselaar, R. M. Schiffelers, and G. Storm, “Liposomal glucocorticoids as tumor-targeted anti-angiogenic nanomedicine in B16 melanoma-bearing mice”, The Journal of Steroid Biochemistry and Molecular Biology, vol. 111, pp. 101–110, 2008.

[134]M. Banciu, R. M. Schiffelers, M. H. Fens, J. M. Metselaar, and G. Storm “Anti-angiogenic effects of liposomal prednisolone phosphate on B16 melanoma in mice”, The Journal of Controlled Release, vol. 113, pp. 1–8, 2006,

[135]M. A. Tran, R. J. Watts, and G. P. Robertson, “Use of liposomes as drug delivery vehicles for treatment of melanoma”, Pigment Cell & Melanoma Research, vol. 22, no. 4, pp. 388–399, 2009.

[136]T. Fujimura, S. Nakagawa, T. Ohtani, Y. Ito, and S. Aiba, “Inhibitory effect of the polyinosinicpolycytidylic acid/cationic liposome on the progression of murine B16F10 melanoma”, The European Journal of Immunology, vol. 36, no.12, pp. 3371–3380, 2006.

[137]W. T. Al-Jamal, K. T. Al-Jamal, P. H. Bomans, P. M. Frederik, and K. Kostarelos, “Functionalized-quantumdot-liposome hybrids as multimodal nanoparticles for cancer”, Small, vol. 4, no.9, pp. 1406–1415, 2008.

[138]G. J. Villares, M. Zigler, and H. Wang, “Targeting melanoma growth and metastasis with systemic delivery of liposome-incorporated protease-activated receptor-1 small interfering RNA”, Cancer Research, vol. 68, no. 21, pp. 9078–9086, 2008.

[139]G. S. Inamdar, S. V. Madhunapantula, and G. P. Robertson, “Targeting the MAPK pathway in melanoma: why some approaches succeed and other fail”, Biochemical Pharmacology, vol. 80, no. 5, pp. 624–637, 2010.

[140]S. Basu, R. Harfouche, S. Soni, G. Chimote, R. A. Mashelkar, and S. Sengupta, “Nanoparticle-mediated targeting of MAPK signaling predisposes tumor to chemotherapy”, Proceedings of the National Academy of Sciences of the United States of America, vol. 106, no. 19, pp. 7957–7961, 2009.

[141]T. X. Xie, F. J. Huang, K. D. Aldape, S. H. Kang, M. Liu, J. E. Gershenwald, K. Xie, R. Sawaya, and S. Huang, “Activation of stat3 in human mela¬noma promotes brain metastasis”, Cancer Research, vol. 66, no. 6, pp. 3188–3196, 2006.

[142]D. Yin, Y. Li, H. Lin, B. Guo, Y. Du, X. Li, H. Jia, X. Zhao, J. Tang, and L. Zhang, “Functional graphene oxide as a plasmid-based Stat3 siRNA carrier inhibits mouse malignant melanoma growth in vivo”, Nanotechnology, vol. 24, no. 10, pp. 105102, 2013.

[143]L. Y. Kong, A. Gelbard, J. Wei, C. Reina-Ortiz, Y. Wang, E. C. Yang, Y. Hailemichael, I. Fokt, A. Jayakumar, W. Qiao, G. N. Fuller, W. W. Overwijk, W. Priebe, and A. B. Heimberger, “Inhibition of p-STAT3 enhances IFN-alpha efficacy against metastatic melanoma in a murine model”, Clinical Cancer Research, vol. 16, no. 9, pp. 2550–2561, 2010.

[144]Y. Chen, S. R. Bathula, Q. Yang, and L. Huang, “Targeted nanoparticles deliver siRNA to melanoma”, The Journal of Investigative Dermatology, vol. 130, no. 12, pp. 2790–2798, 2010.

[145]M. A. Tran, R. Gowda, A. Sharma, E. J. Park, J. Adair, M. Kester, N. B. Smith, and G. P. Robertson, “Targeting V600EB-Raf and Akt3 using nanoliposomal-small interfering RNA inhibits cutaneous melanocytic lesion development”, Cancer Research, vol. 68, no. 18, pp. 7638–7649, 2008.

[146]S. Pizzimenti, E. Ciamporcero, P. Pettazzoni, S. Osella-Abate, M. Novelli, C. Toaldo, M. Husse, M. Daga, R. Minelli, A. Bisazza, P. Ferruti, E. Ranucci, M. Grazia Bernengo, C. Dianzani, F. Biasi, R. Cavalli, G. Barrera, “The inclusion complex of 4-hydroxynonenal with a polymeric derivative of *β*-cyclodextrin enhances the antitumoral efficacy of the aldehyde in several tumor cell lines and in a three-dimensional human melanoma model”, Free Radical Biology and Medicine, vol. 65C, pp. 765-777, 2013.

[147]L. Benimetskaya, K. Ayyanar, N. Kornblum, D. Castanotto, J. Rossi, S. Wu, J. Lai, B. D. Brown, N. Popova, P. Miller, H. McMicken, Y. Chen, and C.A. Stein, “Bcl-2 protein in 518A2 melanoma cells in vivo and in vitro”, Clinical Cancer Research, vol. 12, no. 16, pp. 4940–4948, 2006.

[148]A. Y. Bedikian, M. Millward, H. Pehamberger, R. Conry, M. Gore, U. Trefzer, A. C. Pavlick, R. DeConti, E. M. Hersh, P. Hersey, J. M. Kirkwood, F. G. Haluska, and Oblimersen Melanoma Study Group, “Bcl-2 antisense (oblimersen sodium) plus dacarbazine in patients with advanced melanoma: the Oblimersen Melanoma Study Group”, Journal of Clinical Oncology, vol. 24, no. 29, pp. 4738–4745, 2006.

[149]J. Beloor, C. S. Choi, H. Y. Nam, M. Park, S. H. Kim, A. Jackson, K. Y. Lee, S. W. Kim, P. Kumar, and S. K. Lee “Arginine-engrafted biodegradable polymer for the systemic delivery of therapeutic siRNA”, Biomaterials, vol. 33, no. 5, pp. 1640–1650, 2012.

[150]P. A. Ott, J. Chang, K. Madden, R. Kannan, C. Muren, C. Escano, X. Cheng, Y. Shao, S. Mendoza, A. Gandhi, L. Liebes, and A. C. Pavlick, “Oblimersen in combination with temozo¬lomide and albumin-bound paclitaxel in patients with advanced melanoma: a phase I trial”, Cancer Chemotherapy and Pharmacology, vol. 71, no. 1, pp. 183–191, 2013.

[151]Z. Zhang, S. Tongchusak, Y. Mizukami, Y. J. Kang, T. Ioji, M. Touma, B. Reinhold, D. B. Keskin, E. L. Reinherz, and T. Sasada, “Induction of anti-tumor cytotoxic T cell responses through PLGA-nanoparticle mediated antigen delivery”, Biomaterials, vol. 32, no. 14, pp. 3666–3678, 2011.

[152]F. Perche, T. Benvegnu, M. Berchel, L. Lebegue, C. Pichon, P.A. Jaffrès, and P. Midoux, “Enhancement of dendritic cells transfection in vivo and of vaccination against B16F10 melanoma with mannosylated histidylated lipopolyplexes loaded with tumor antigen messenger RNA”, Nanomedicine, vol. 7, no. 4, pp. 445–453, 2011.

[153]H. Yao, S. S. Ng, L. F. Huo, B. K. Chow, Z. Shen, M. Yang, J. Sze, O. Ko, M. Li, A. Yue, L. W. Lu, X. W. Bian, H. F. Kung, and M. C. Lin, “Effective melanoma immunotherapy with interleukin-2 delivered by a novel polymeric nanoparticle”, Molecular Cancer Therapeutics, vol. 10, no. 6, pp. 1082–1092, 2011.

[154]H. He, V. Grignol, V. Karpa, C. Yen, K. LaPerle, X. Zhang, N. B. Jones, M. I. Liang, G. B. Lesinski, W. S. Ho, W. E. Carson 3rd, and L. J. Lee, “Use of a nanoporous biodegradable miniature device to regulate cytokine release for cancer treatment”, The Journal of Controlled Release, vol. 151, no. 3, pp. 239–245, 2011.

[155]D. E. Speiser, K. Schwarz, P. Baumgaertner, V. Manolova, E. Devevre, W. Sterry, P. Walden, A. Zippelius, K. B. Conzett, G. Senti, V. Voelter, J. P. Cerottini, D. Guggisberg, J. Willers, C. Geldhof, P. Romero, T. Kündig, A. Knuth, R. Dummer, U. Trefzer, and M. F. Bachmann “Memory and effector CD8 T-cell responses after nanoparticle vaccination of melanoma patients”, Journal of Immunotherapy, vol. 33, no. 8, pp. 848–858, 2010.

[156]M. Camerin, M. Magaraggia, M. Soncin, G. Jori, M. Moreno, I. Chambrier, M. J. Cook, and D. A. Russell “The in vivo efficacy of phthalocyanine-nanoparticle conjugates for the photodynamic therapy of amelanotic melanoma”, The European Journal of Cancer, vol. 46, no. 10, pp. 1910–1918, 2010.

[157]M. Sato, T. Yamashita, M. Ohkura, Y. Osai, A. Sato, T. Takada, H. Matsusaka, I. Ono, Y. Tamura, N. Sato, Y. Sasaki, A. Ito, H. Honda, K. Wakamatsu, S. Ito, and K. Jimbow “N-propionyl-cysteaminylphenol-magnetite conjugate (NPrCAP/M) is a nanoparticle for the targeted growth suppression of melanoma cells”, Journal of Investigation Dermatology, vol. 129, no 9, pp. 2233-2241, 2009.

## Figures and Tables

**Figure 1:**
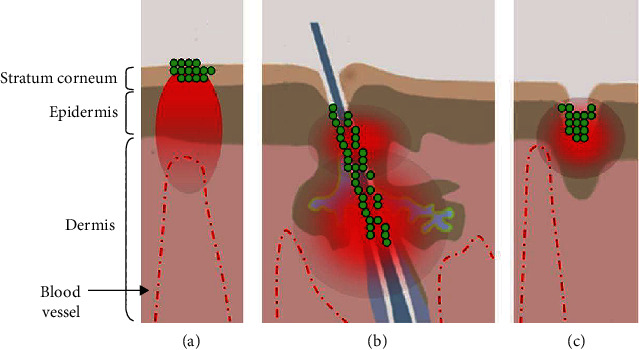
Sites in the skin for nanoparticle delivery. Topical nanoparticle drug delivery takes place in three major sites: stratum corneum (SC) surface (a), openings of hair follicles (infundibulum) (b), and furrows (dermatoglyphics) (c). The nanoparticles are shown in green and the drug in red. Other sites for delivery are the viable epidermis and dermis. Modified by Prow et al. [68].
